# Cell‐free DNA as a biomarker of aging

**DOI:** 10.1111/acel.12890

**Published:** 2018-12-20

**Authors:** Yee Voan Teo, Miriam Capri, Cristina Morsiani, Grazia Pizza, Ana Maria Caetano Faria, Claudio Franceschi, Nicola Neretti

**Affiliations:** ^1^ Department of Molecular Biology, Cell Biology and Biochemistry Brown University Providence Rhode Island; ^2^ CIG Interdepartmental Centre “Galvani” University of Bologna Bologna Italy; ^3^ DIMES‐Department of Experimental, Diagnostic and Specialty Medicine University of Bologna Bologna Italy; ^4^ Section of Cell Biology and Functional Genomics, Division of Diabetes, Endocrinology & Metabolism, Department of Medicine Imperial College London London UK; ^5^ Departamento de Bioquímica e Imunologia, Instituto de Ciências Biológicas Universidade Federal de Minas Gerais Belo Horizonte Brazil; ^6^ IRCCS, Institute of Neurological Sciences of Bologna Bologna Italy; ^7^ Center for Computational Molecular Biology Brown University Providence Rhode Island

**Keywords:** aging, cell‐free DNA, epigenetics

## Abstract

Cell‐free DNA (cfDNA) is present in the circulating plasma and other body fluids and is known to originate mainly from apoptotic cells. Here, we provide the first in vivo evidence of global and local chromatin changes in human aging by analyzing cfDNA from the blood of individuals of different age groups. Our results show that nucleosome signals inferred from cfDNA are consistent with the redistribution of heterochromatin observed in cellular senescence and aging in other model systems. In addition, we detected a relative cfDNA loss at several genomic locations, such as transcription start and termination sites, 5′UTR of L1HS retrotransposons and dimeric AluY elements with age. Our results also revealed age and deteriorating health status correlate with increased enrichment of signals from cells in different tissues. In conclusion, our results show that the sequencing of circulating cfDNA from human blood plasma can be used as a noninvasive methodology to study age‐associated changes to the epigenome in vivo.

## 1 INTRODUCTION

During the last century, human life expectancy has more than doubled, resulting in a large worldwide increase in the elderly population: The number of individuals aged 80 or over is projected to triple by 2050 and to increase to nearly seven times by 2100 (United Nations, 2017), and the number of centenarians is also expected to increase globally. Because increased longevity does not necessarily translate into increased healthspan, improving the latter is an urgent priority. Hence, biomarkers of aging that can be translated to the clinical setting are of particular interest.

Several aging biomarkers such as C‐reactive protein and insulin‐like growth factor‐1 have been identified as predictive for mortality (Castagne et al., 2018). The identification of circulating biomarkers is of increasing interest in the study of human aging, especially when these biomarkers are applied to the measurement of biological age (Capri et al., 2015). Recent data showed that subjects of the same chronological age, including centenarians, can have younger or older biological ages that, in turn, are associated with morbidity and mortality (Chen et al., 2016). Among the different biomarkers that have been proposed, which include DNA methylation and *N*‐glycans (Horvath, 2013; Miura & Endo, 2016), cell‐free DNA (cfDNA) appears particularly promising due to the ease of collecting specimens and the ever‐decreasing costs of genomic sequencing. However, little is known about how cfDNA changes with age.

High levels of cfDNA were first reported in 1966 in the circulating serum of patients with systemic lupus erythematosus (Tan, Schur, Carr, & Kunkel, 1966) and were later discovered in the plasma of cancer patients (Stroun, Anker, Lyautey, Lederrey, & Maurice, 1987). cfDNA originates primarily from cell death through apoptosis or necrosis (van der Vaart & Pretorius, 2007), and recently, new methods have been developed to trace the tissues of origin of cfDNA through nucleosome positioning and methylation footprints (Lehmann‐Werman et al., 2016; Snyder, Kircher, Hill, Daza, & Shendure, 2016). These methodologies allow the detection of tissue‐specific damage or disease through liquid biopsies.

The aging process is associated with cellular stress and is accompanied by alterations to the number of apoptotic cells and DNA release (Jylhava et al., 2013; Pollack, Phaneuf, Dirks, & Leeuwenburgh, 2002). Older individuals were reported to exhibit higher levels of circulating cfDNA (Jylhava et al., 2011), including mitochondrial DNA (cf‐mtDNA) (Pinti et al., 2014). Aging is also associated with chronic systemic inflammation or inflammaging. The cause of this phenomenon in older individuals may come from different sources, one of which is the increased number of senescent cells that can secrete senescence‐associated secretory phenotype factors to drive inflammation (Franceschi & Campisi, 2014). Other factors such as age‐associated accumulation of metabolites or cell debris, including self and non‐self‐nucleic acids (Franceschi, Garagnani, Vitale, Capri, & Salvioli, 2017), can act as damage‐associated molecular patterns (DAMPs) that trigger immune response and subsequent inflammation (Franceschi & Campisi, 2014). For instance, high level of total cfDNA in nonagenarians is associated with systemic inflammation and frailty (Jylhava et al., 2013). cfDNA has also been shown to be one of the triggers to adipocyte inflammation in obese mice due to the increased cell death in fat tissues (Nishimoto et al., 2016).

In this study, we used cfDNA to characterize the nucleosome landscape and the contributing tissues of age‐associated cell death. We performed whole‐genome sequencing on cfDNA collected from the plasma of individuals in four groups composed of young, old, and two cohorts of centenarians (divided into healthy and unhealthy populations).

## 2 RESULTS

### 2.1 Profiling of cfDNA at different ages and in extreme longevity

Aging is known to be associated with increased cell death, which may contribute to a change in the cfDNA released into circulation. Hence, we profiled cfDNA extracted from a total of 12 individuals from different ages and health conditions. Specifically, we performed whole‐genome sequencing of cfDNA from three 25‐year‐old ± 0.5 (*SD*) subjects (referred to as young), three 71‐year‐old ± 1.6 subjects (referred to as old), and six 101.8‐year‐old ± 1.1 centenarians (Supporting Information Tables S1 and S2). The centenarian cohort was further divided into two groups: three healthy and three unhealthy individuals. Healthy centenarians were characterized as having good cognitive performance, that is, SMMSE (Standardized Mini‐Mental State Examination) > 24 scores, retaining the ability to walk, and having a high ADL (Activities of Daily Living) score (Supporting Information Table S1). In contrast, unhealthy centenarians had dementia, were not able to perform the SMMSE, and were bedridden. In addition, the two centenarian cohorts showed significant differences in 5 out of 32 hemato‐biochemical parameters tested: RBC, HGB, HCT, ALB, and HDL (Supporting Information Table S5).

### 2.2 Cell‐free DNA reveals in vivo nucleosome landscape changes with age

**Figure 1:**
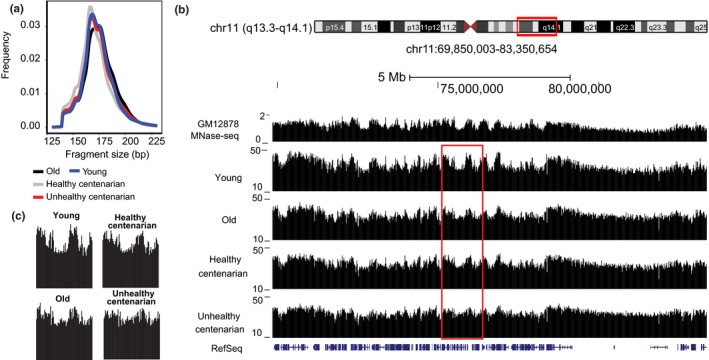
Cell‐free DNA (cfDNA) signals reveal the nucleosome landscape of cells. (a) Fragment size of plasma cfDNA from different age groups. (b) Genome browser view of MNase‐seq data from GM12878 lymphoblastoid cell line and cfDNA signals from young, old, and centenarians in chr 11. (c) A close‐up view of the boxed region in (b) shows the redistribution of cfDNA signals with age

The analysis of the fragment length of cfDNA derived from blood plasma showed an enrichment of 166‐ to 175‐bp fragments (Figure 1a, Supporting Information Table S3), which corresponds to the length of a chromatosome. In healthy individuals, most of these cfDNA fragments originate from apoptotic cells of hematopoietic origin (Lo et al., 2010). Several studies have demonstrated that the fragmentation patterns of cfDNA can reveal the nucleosome landscape of dead cells from which the cfDNA is derived. This is because DNA wrapped around the histone octamers and linker histones is protected from digestion during apoptosis (Ivanov, Baranova, Butler, Spellman, & Mileyko, 2015; Snyder et al., 2016).

**Figure 2:**
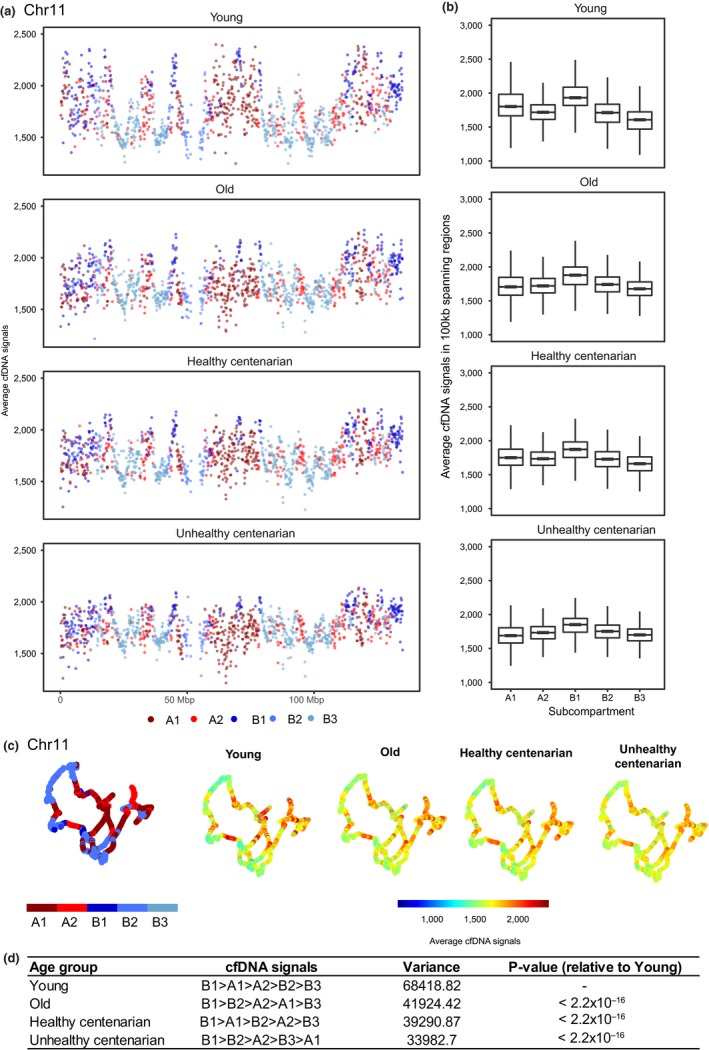
Cell‐free DNA (cfDNA) signals in different subcompartments. (a) Average cfDNA signals of 100‐kb regions spanning across chr 11 in each age group. Colors represent the subcompartment that these 100‐kb spanning regions are in based on GM12878 Hi‐C data. The 3D organization of chr 11 at 100 kb resolution were colored by either the subcompartments or the average cfDNA signals. (b) Boxplots showing the average cfDNA signals in 100‐kb spanning regions in all chromosomes across subcompartments. The top and bottom bounds of the boxplot correspond to the 75th and 25th percentiles, respectively. (c) The 3D organization of chr 11 at 100 kb resolution, colored by the average cfDNA signals or the subcompartment regions. (d) Quantitation of cfDNA signals in all chromosomes across subcompartments (*p* < 0.005; one‐way ANOVA and Tukey's test)

We used DANPOS2 to identify the cfDNA signals and to define the nucleosome landscape in these samples (Chen et al., 2013). We compared the nucleosome positioning patterns to micrococcal nuclease‐seq (MNase‐seq) nucleosome signals of GM12878, a lymphoblastoid cell line, obtained from ENCODE (Consortium, 2012). The signals from cfDNA aligned well with the nucleosome signals from GM12878 (Pearson's = 0.77, *p* < 2.2 × 10^−16^), suggesting similarity between the fragmentation patterns of cfDNA and MNase‐treated samples (Figure 1b, Supporting Information Figure S1a,c). Notably, signals observed in young individuals are smoothened with age, with most of the regions showing a redistribution of the cfDNA signal (Figure 1b,c, Supporting Information Figure S1a,b). The signals became less pronounced, especially in the unhealthy centenarians. To quantify and characterize this age‐dependent change on a global scale, we analyzed the average cfDNA signals within 100‐kb regions across the whole genome and annotated the signals with subcompartments identified from the GM12878 cell line (Rao et al., 2014; Figure 2a–c). These subcompartments were identified using Hi‐C data and were associated with distinct histone modifications. Subcompartments A1 and A2 consist of euchromatic regions that are gene rich, subcompartment B1 consists of facultative heterochromatic regions, subcompartment B2 is enriched at the nuclear lamina and at nucleolus‐associated domains (NADs), whereas subcompartment B3 is also enriched at the nuclear lamina but not at NADs. We excluded subcompartment B4 because it is only present on chromosome 19 (Rao et al., 2014). In young individuals, cfDNA signals are the highest in subcompartment B1, followed by compartment A and subcompartments B2 and B3 (Rao et al., 2014) (*p* < 2 × 10^−16^; Kruskal–Wallis test and post hoc Dunn's test) (Figure 2a–d). We also applied the same method to GM12878 MNase‐seq and observed the same pattern of signal enrichment in the different subcompartments to the cfDNA signals, further supporting the similarity between MNase‐treated and cfDNA samples (*p* < 2 × 10^−16^; Kruskal–Wallis test and post hoc Dunn's test) (Supporting Information Figure S2a,b). In contrast, we analyzed ATAC‐seq data from GM12878 in the different subcompartments and observed higher signals in compartment A and lower signals in compartment B, including B1, suggesting that the highest signals observed previously in subcompartment B1 is a unique feature of both MNase‐seq and cfDNA‐seq (Supporting Information Figure S2c).

**Figure 3:**
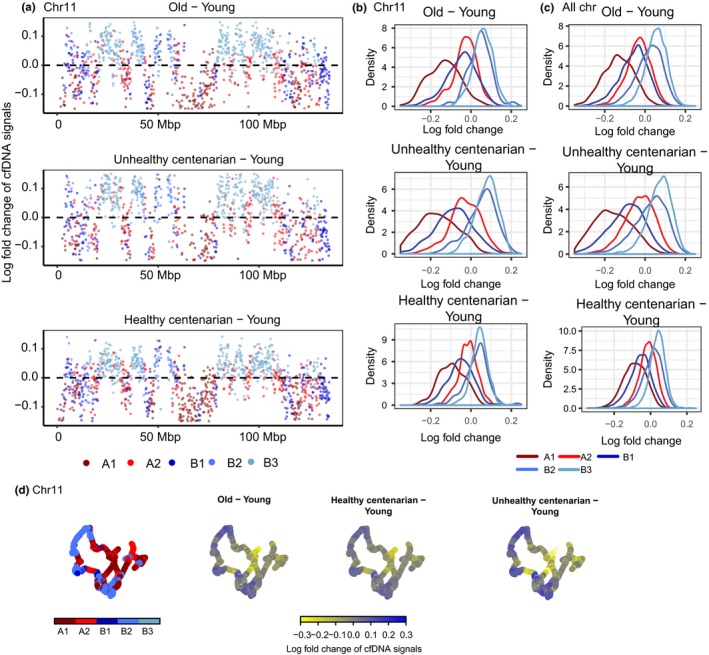
Differential cell‐free DNA (cfDNA) signals reveal a loss of signals in compartment A1 and B1 and a gain of signals in compartment B2 and B3 with age. (a) Log fold change of cfDNA signals with 100 kb spanning across chr 11 of each age group relative to young. Colors represent the compartment that these 100‐kb spanning regions are in based on GM12878 Hi‐C data. (b) The distribution of log fold change of cfDNA signals by compartments in chr 11. (c) The distribution of log fold change of cfDNA signals by compartments in all chromosomes. (d) The 3D organization of chr 11 at 100 kb resolution, colored by the log fold change of cfDNA signals

**Figure 4:**
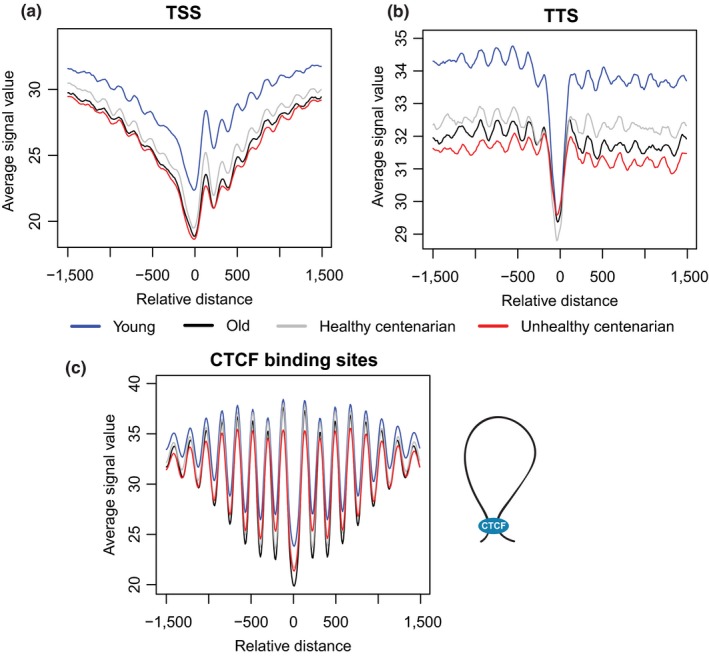
Local nucleosome profiles’ changes with age. (a) Average cell‐free DNA signals at transcription start sites, (b) transcription termination sites, and (c) CCCTC‐binding factor (CTCF) showed an expected NDR flanked by well‐positioned nucleosomes, but these signals attenuate with age. CTCF has been shown to play roles in mediating the formation of chromatin loops

**Figure 5:**
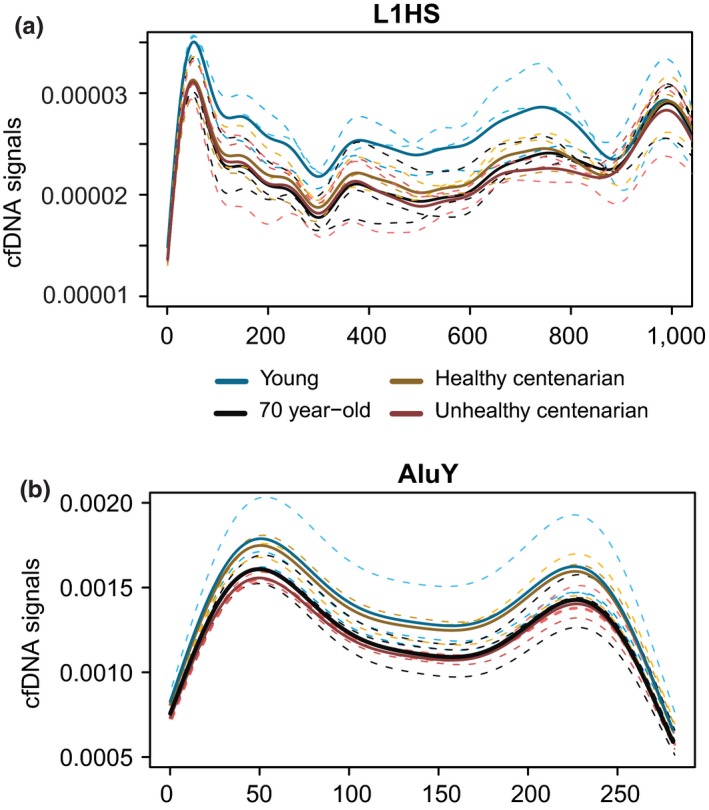
Cell‐free DNA (cfDNA) signals at repetitive elements. (a) cfDNA at the first 1,000 bp of L1HS consensus sequence. The dotted lines indicate replicates in different age groups, and the solid lines mark the average of cfDNA signals in the respective age group. (b) cfDNA signals in dimeric AluY. The dotted lines indicate replicates in different age groups, and the solid lines mark the average of cfDNA signals in the respective age group

**Figure 6:**
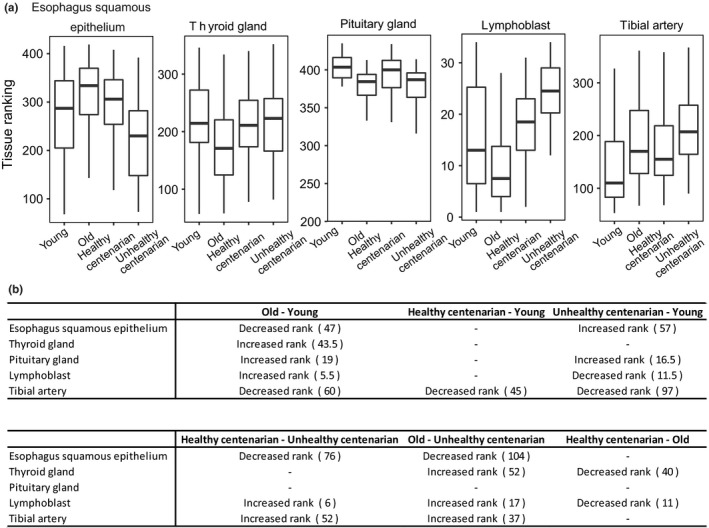
Cell‐free DNA origin changes with age. (a) Boxplots showing tissues that showed significant rank changes between age groups. The top and bottom bounds of the boxplot correspond to the 75th and 25th percentiles, respectively. (b) Significant change in ranking is shown in the table, with the magnitude of tissue ranking change indicated by numbers in parentheses (*p*‐value < 0.05, Kruskal–Wallis test and post hoc Dunn's test)

The variance of cfDNA signals across compartments significantly decreased with age (Levene's test, all pairwise comparisons *p* < 0.05) and is the lowest in the unhealthy centenarians, further supporting the redistribution of signals from heterochromatin regions to euchromatic regions in old age and deteriorating health condition (Figure 2d). Overall, we observed a significant increase in cfDNA signals in subcompartments B2 and B3 and decrease in subcompartments A1, A2, and B1 in the old group, healthy centenarians (except for A2), and unhealthy centenarians compared to young group (Figure 3a–d, Table 1). Significantly lower cfDNA signals were also observed in subcompartments A1 and A2, and significantly higher cfDNA signals were observed in subcompartments B2 and B3 in unhealthy centenarians and in the old group compared to healthy centenarians. This trend was similar to the comparison to the young group. (Supporting Information Figure S3a–d, Table 1). The comparison between unhealthy centenarians and the old group showed increased signals in subcompartments A2, B2, and B3 and decreased signals in subcompartments A1 and B1 in unhealthy centenarians. The global cfDNA signals of unhealthy centenarians are highly correlated to old and differed the most from the young group (Supporting Information Figure S3e,f). Healthy centenarian displayed the highest cfDNA signals correlation among other age groups when compared to the young group (Pearson's correlation = 0.603).

**Table 1:** Change of cell‐free DNA signals across different age groups. The change in direction is only identified for significant comparisons in which zero is excluded from the credible interval

	Comparisons	Mean of fold change	Difference in posterior median	Credible interval	Change in direction	*p*‐Value
A1	Healthy centenarian–old	0.0448	0.0282	0.0268, 0.0298	Increased signal	<0.0001
	Healthy centenarian–unhealthy centenarian	0.0760	0.0478	0.0461, 0.0494	Increased signal	<0.0001
	Healthy centenarian–young	−0.0871	−0.0472	−0.0487, −0.0455	Decreased signal	<0.0001
	Old–unhealthy centenarian	0.0312	0.0196	0.018, 0.0212	Increased signal	<0.0001
	Old–young	−0.1319	−0.0754	−0.0769, −0.0738	Decreased signal	<0.0001
	Unhealthy centenarian–young	−0.1631	−0.095	−0.0967, −0.0933	Decreased signal	<0.0001
A2	Healthy centenarian–old	0.0177	0.009	0.0082, 0.01	Increased signal	<0.0001
	Healthy centenarian–unhealthy centenarian	0.0130	0.0045	0.0037, 0.0054	Increased signal	<0.0001
	Healthy centenarian–young	−0.0166	−0.0006	−0.0015, 0.0003	—	0.5613
	Old–unhealthy centenarian	−0.0047	−0.0045	−0.0054, 0.0036	Decreased signal	<0.0001
	Old–young	−0.0343	−0.0096	−0.0104, −0.0086	Decreased signal	<0.0001
	Unhealthy centenarian–young	−0.0297	−0.0051	−0.006, −0.0041	Decreased signal	<0.0001
B1	Healthy centenarian–old	−0.0004	−0.0034	−0.0049, −0.0018	Decreased signal	<0.0001
	Healthy centenarian–unhealthy centenarian	0.0338	0.0185	0.0171, 0.02	Increased signal	<0.0001
	Healthy centenarian–young	−0.0591	−0.0288	−0.0303, −0.0274	Decreased signal	<0.0001
	Old–unhealthy centenarian	0.0342	0.0218	0.0204, 0.0233	Increased signal	<0.0001
	Old–young	−0.0587	−0.0255	−0.027, −0.0241	Decreased signal	<0.0001
	Unhealthy centenarian–young	−0.0928	−0.0474	−0.0487, −0.0459	Decreased signal	<0.0001
B2	Healthy centenarian–old	−0.0118	−0.0112	−0.0125, −0.0099	Decreased signal	<0.0001
	Healthy centenarian–unhealthy centenarian	−0.0181	−0.0169	−0.0183, −0.0156	Decreased signal	<0.0001
	Healthy centenarian–young	0.0086	0.0169	0.0156, 0.0183	Increased signal	<0.0001
	Old–unhealthy centenarian	−0.0063	−0.0057	−0.0071, −0.0044	Decreased signal	<0.0001
	Old–young	0.0204	0.0281	0.0268, 0.0296	Increased signal	<0.0001
	Unhealthy centenarian–young	0.0267	0.0338	0.0326, 0.0351	Increased signal	<0.0001
B3	Healthy centenarian–old	−0.0163	−0.0143	−0.015, −0.0135	Decreased signal	<0.0001
	Healthy centenarian–unhealthy centenarian	−0.0326	−0.027	−0.0278, −0.0262	Decreased signal	<0.0001
	Healthy centenarian–young	0.0376	0.037	0.0363, 0.0379	Increased signal	<0.0001
	Old–unhealthy centenarian	−0.0163	−0.0128	−0.0135, −0.012	Decreased signal	<0.0001
	Old–young	0.0539	0.0513	0.0505, 0.0520	Increased signal	<0.0001
	Unhealthy centenarian–young	0.0702	0.0641	0.0632, 0.0648	Increased signal	<0.0001

### 2.3 Local cfDNA profiles with age

To study local nucleosome profile changes with age in gene regions, we computed the average cfDNA signals across all genes relative to transcription start sites (TSS) and transcription termination sites (TTS) of genes in autosomal chromosomes (Figure 4a,b). We observed the typical nucleosome‐depleted regions (NDRs) at TSS and TTS, which are also commonly observed in MNase assays (Schones et al., 2008; Valouev et al., 2011). Notably, within the *±*1,500 bp range from TSS, we observed a relative loss of cfDNA signals with age. We detected the highest cfDNA signals in young, followed by healthy centenarian, old, and, lastly, unhealthy centenarians (Figure 4a, Supporting Information Figure S4a) (Kruskal–Wallis test [*p* < 2.2 × 10^−16^] with Dunn's post hoc test: unhealthy centenarian and young, *p* = 7.4 × 10^−112^; old and young, *p* = 2.2 × 10^−91^; healthy centenarian and young; *p* = 1.3 × 10^−51^, old and unhealthy centenarian; *p* = 0.026, healthy centenarian and unhealthy centenarian, *p* = 2.11 × 10^−13^; healthy centenarian and old, *p* = 2.8 × 10^−7^). To identify the variability within age groups, we also calculated the coefficient of variation (CV) of cfDNA signals at the TSS. Young replicates showed a 6.2% CV, old replicates showed a 5.1% CV, healthy centenarian replicates showed a 4% CV, and unhealthy centenarian replicates showed a 5% CV. In addition, we also observed a similar decrease in signals within the *±*1,500 bp range from TTS with age (Figure 4b, Supporting Information Figure S4b) (Kruskal–Wallis test [*p* < 2.2 × 10^−16^] with Dunn's post hoc test: unhealthy centenarian and young, *p* = 2.1 × 10^−213^; old and young, *p* = 6.4 × 10^−93^; healthy centenarian and young, *p* = 7.8 × 10^−38^; old and unhealthy centenarian, *p* = 8.4 × 10^−27^; healthy centenarian and unhealthy centenarian, *p* = 7.1 × 10^−75^; healthy centenarian and old, *p* = 2.7 × 10^−14^). Young replicates showed a 5.4% CV, old replicates showed a 2.4% CV, healthy centenarian replicates showed a 2.2% CV, and unhealthy centenarian replicates showed a 1.5% CV. Global nucleosome loss has been reported in aging yeast, and this phenomenon leads to the deregulation of transcriptional activity with age (Hu et al., 2014).

In addition to TSS and TTS, we also assess cfDNA signals around CCCTC‐binding factor (CTCF)‐binding sites, which were obtained from GM12878 ENCODE ChIP‐Seq data. CTCF has been shown to play roles in several biological processes, including the regulation of 3D chromatin structure in cells, mediating the formation of chromatin loops and the demarcation of heterochromatic and euchromatic regions (Figure 4c) (Ong & Corces, 2014). CTCF‐binding sites are also known to be flanked by well‐positioned nucleosomes (Fu, Sinha, Peterson, & Weng, 2008). We observed NDR at the center of CTCF‐binding sites and strong oscillatory signals immediately flanking the region (Figure 4c, Supporting Information Figure S4c). This feature has been reported in different cell types, such as human T cells, K562, and GM12878 cell lines (Chen, Tian, Shu, Bo, & Wang, 2012; Fu et al., 2008). Unlike TSS and TTS, we observed decreased signals in all comparisons but not between old and unhealthy centenarians (Figure 4c, Supporting Information Figure S3c) (Kruskal–Wallis test [*p* < 2.2 × 10^−16^] with Dunn's post hoc test: unhealthy centenarian and young, *p* = 9.7 × 10^−33^; old and young, *p* = 1.3 × 10^−25^; healthy centenarian and young, *p* = 3.7 × 10^−12^; old and unhealthy centenarian, *p* = 0.13; healthy centenarian and unhealthy centenarian, *p* = 7.8 × 10^−7^; healthy centenarian and old, *p* = 5.1 × 10^−4^). Young replicates showed a 3.7% CV, old replicates showed a 2.7% CV, healthy centenarian replicates showed a 1.4% CV, and unhealthy centenarian replicates showed a 3.6% CV.

To assess the contribution of TSS and TTS to the global change of cfDNA distribution and local nucleosome profile, we masked ±1,500 bp of all TSS and TTS from the genome and reanalyzed the changes of cfDNA signals in subcompartments. We still observed a loss of signals in compartment A and subcompartment B1 and a gain of signals in compartment B2 and B3 in comparison with young (Supporting Information Figure S4d, Table S4). Therefore, the local change of TSS and TTS is not a significant contributing factor to the change in global chromatin accessibility.

### 2.4 cfDNA signals at transposable elements

We observed decreased cfDNA signals with age within the first 668 bp of the 5′UTR of a transposable element, L1HS, in which the promoter and enhancer were found (Speek, 2001; Swergold, 1990), with the lowest and comparable signals in old individuals and unhealthy centenarians (Figure 5a, Supporting Information Figure S5a) (Kruskal–Wallis test [*p* < 2.2 × 10^−16^] with Dunn's post hoc test: unhealthy centenarian and young, *p* = 6.8 × 10^−301^; old and young, *p* < 0.0001; healthy centenarian and young, *p* = 7.4 × 10^−191^; old and unhealthy centenarian, *p* = 0.48; healthy centenarian and unhealthy centenarian, *p* = 3.2 × 10^−14^; healthy centenarian and old, *p* = 1.3 × 10^−16^). To identify the variability within age groups, we also calculated the CV of cfDNA signals at the 5′UTR of L1HS, in which young replicates showed a 6.9% CV, old replicates showed a 11.1% CV, healthy centenarian replicates showed a 8.2% CV, and unhealthy centenarian replicates showed a 10.97% CV.

In addition, we also analyzed the signals in another transposable element, AluY. Healthy centenarians and young individuals showed very similar cfDNA coverage at AluY (*p* = 0.67), whereas unhealthy centenarians and old individuals showed lower cfDNA signals compared to young (Figure 5b) (*p* = 1 × 10^−52^ and *p* = 1.2 × 10^−35^, respectively). The CV of AluY signals is 14.4% for young, 8.3% for old, 8.3% for healthy centenarian replicates, and 3.2% for unhealthy centenarian. Transposable elements were previously found to be derepressed in aging and cancer (Anwar, Wulaningsih, & Lehmann, 2017; Criscione, Zhang, Thompson, Sedivy, & Neretti, 2014; De Cecco, Criscione, Peckham, et al., 2013; De Cecco, Criscione, Peterson, et al., 2013), which is possibly reflected in the cfDNA footprints. Hence, we also analyzed cfDNA samples from three prostate cancer patients, a liver cancer patient, and a healthy individual from Snyder et al. (2016) to profile the cfDNA coverage at L1HS and AluY. We observed the lowest cfDNA signals at the 5′UTR of L1HS retrotransposon in the liver cancer patient, followed by the prostate cancer samples (Kruskal–Wallis test [*p* < 2.2 × 10^−16^] with Dunn's post hoc test: liver cancer and healthy, *p* = 9.4 × 10^−51^; liver cancer and prostate cancer, *p* = 5.5 × 10^−16^; healthy and prostate, *p* = 7 × 10^−25^). The healthy individual displayed the highest cfDNA coverage (Supporting Information Figure S5b,c) at the 5′UTR of L1HS. We also observed the highest signals at AluY in the healthy individual, followed by liver cancer and prostate cancer (Supporting Information Figure S5d) (Kruskal–Wallis test [*p* < 2.2 × 10^−16^] with Dunn's post hoc test: liver cancer and healthy, *p* = 4.9 × 10^−14^; liver cancer and prostate cancer, *p* = 7.9 × 10^−21^; healthy and prostate, *p* = 5.2 × 10^−77^).

### 2.5 Increased cfDNA from tissues in old individuals and unhealthy centenarians

A significant portion of cfDNA is derived from the hematopoietic lineage. However, deregulations of apoptosis have been implicated in aging as its rate increases in some cell types (Ciccocioppo et al., 2002; Tower, 2015; Vazquez‐Padron et al., 2004). To identify the tissues that give rise to this cfDNA in young, old, and centenarians, we used the method developed by Snyder et al. (2016) in calculating window protection score (WPS) and performing fast Fourier transformation (FFT) to correlate the corresponding intensities with the gene expression values of tissues obtained from the Genotype‐Tissue Expression Project (GTEx). Specifically, we used gene expression values from individuals of two different age groups, 20–24 years old and 66–70 years old, to avoid the bias of using young or old gene expression to correlate with centenarians’ cfDNA intensities. Subsequently, we performed Kruskal–Wallis test to identify significant changes in tissue rankings (Supporting Information Figure S6a).

We applied this method to the lung cancer dataset from Snyder et al. (2016). We detected that liver tissues from GTEx significantly increased in rank (*p* < 0.05) in the liver cancer sample compared to the two healthy controls, suggesting an increased contribution of cfDNA from the liver (Supporting Information Figure S6c) and verifying the method using the GTEx dataset.

By analyzing the aging cfDNA data, we detected the highest correlation between the FFT intensities and gene expression values for each tissue in the 193–199 bp frequency range, which is also consistent with the finding from Snyder et al. (2016) (Supporting Information Figure S6b). We observed that healthy centenarians did not show any detectable increased tissues’ cell death in comparison with the young group, whereas old individuals and unhealthy centenarians showed increased contributing tissues compared to young individuals (Figure 6a,b).

## 3 DISCUSSION

Cell‐free DNA has gained much attention in recent years for its translational potential as a biomarker for cancer (Jung, Fleischhacker, & Rabien, 2010), acute organ transplant rejection (De Vlaminck et al., 2015), and aneuploidy maternal screening tests for genetic disorders like Down syndrome (Ke, Zhao, & Wang, 2015). Here, we studied cfDNA via DNA sequencing in individuals of different aged and health conditions. cfDNA has been reported to increase with age and in nonagenarians (Jylhava et al., 2013; Pinti et al., 2014). However, we did not observe any significant difference in the concentration of cfDNA in our samples across groups, due to the high interindividual variability within groups (1‐way ANOVA, *p* = 0.8; Supporting Information Table S1). It is important to note that inter‐individual variation in biological age markers, such as DNA methylation and potentially age‐associated proteins or transcript biomarkers, is usually high (Franceschi et al., 2018; Horvath, Garagnani, et al., 2015; Horvath, Pirazzini, et al., 2015; Kooman et al., 2017; Kooman, Kotanko, Schols, Shiels, & Stenvinkel, 2014). This is in accordance with the concept of “immunobiography,” which stipulates that high heterogeneity is expected particularly in old populations, as individuals may have accelerated or decelerated aging processes (Franceschi, Salvioli, et al., 2017). Therefore, it is important to study cfDNA in the plasma of individuals in very different age groups, including centenarians, a group that has reached the extreme limit of human lifespan (Arosio et al., 2017; Ostan et al., 2018). As of January 1st 2018, the number of centenarians in Italy was 15,647 (83% females) out of a resident population of 60,483,973 inhabitants, i.e. a ratio of 1:3865 (ISTAT, report on Italian population released on September 6th, 2018, https://www.istat.it/it/files//2018/09/Report_popolazione_residente_e_stato_civile.pdf). In our dataset of Italian centenarians (unpublished), those with an optimal and unimpaired cognitive status were 21.6% (SMMSE 24‐30), while those with worse cognitive status (unable to perform the SMMSE test) were 23.2%. In addition, centenarians with the best physical status were 17.5% (ADL = 5) while those in worse status (ADL 0‐2) were 66.5%. In this study, we included centenarians of extreme phenotypes, i.e. centenarians who have both optimal cognitive and physical status or vice versa.

Deep sequencing of cfDNA with currently available DNA sequencing technologies has never been applied to human aging and longevity. This approach can be used to identify possible aging markers and to investigate basic molecular mechanisms of aging, such as the changes of chromatin structure as cells age. For example, yeast aging is associated with the loss of nucleosomes (Hu et al., 2014) and replicative senescence is associated with increased accessibility of heterochromatic regions (Criscione, Teo, & Neretti, 2016; De Cecco, Criscione, Peckham, et al., 2013). So far, these genome‐wide chromatin reorganizations have only been observed in model organisms and cell cultures.

Here, we provide comprehensive in vivo evidences of global and local chromatin changes in human aging. We observed the highest cfDNA signal in subcompartment B1, followed by A1, A2, B2, and, lastly, B3, in young individual, consistent with the MNase‐seq result of GM12878 lymphoblastoid cell line. The signal from B1, which is a facultative heterochromatin subcompartment, behaves differently than that of constitutive heterochromatin. It has been previously shown that in HeLa cells, facultative heterochromatin bordering euchromatin has higher MNase‐seq signal, which is consistent with our findings (Li & Zhou, 2013). However, when we analyzed ATAC‐seq signals in different subcompartments, all B subcompartments, including B1, showed lower signals than any of the A subcompartments. One way to explain this unique feature of B1 is a different accessibility of facultative heterochromatin to the enzymes used in the three assays. MNase is a 17‐kDa protein (Taniuchi, Anfinsen, & Sodja, 1967) and caspase‐activated DNase (CAD) is a 40‐kDa protein (Liu et al., 1998). Both are smaller in size than the Tn5 transposase (53.3 kDa) (Naumann & Reznikoff, 2002) and the adapters that it carries. Therefore, it is possible that MNase and CAD can more easily access and cut DNA in the facultative heterochromatin than the larger size transposase. On the other hand, B2 and B3 consisting of constitutive heterochromatin are the least accessible for all three enzymes, hence, display the lowest signals among all subcompartments. This is consistent with the result of a recent study that shows that different MNase titration is required to explain chromatin accessibility because nucleosome signals vary with different level of digestion (Mieczkowski et al., 2016).

We observed increased cfDNA signals in subcompartments B2 and B3, which are enriched in lamina‐associated domains (LADs), with age. Specifically, cfDNA levels from subcompartment B2 in old individuals and centenarian resemble that of subcompartment A1 in young individuals, suggesting that B2 has become more euchromatic. In addition, subcompartment A1 in old becomes more similar to subcompartment B2 in young. This indicates that there is a switch between subcompartments A1 and B2 with age. The decrease in cfDNA signals in subcompartment A1 in unhealthy centenarians leads to the subcompartment having lower signals compared to subcompartments A2, B2, and B3, indicating that it has become the most heterochromatic subcompartment. Although signals from subcompartment B1 decreased with age, it remains the subcompartment with the highest cfDNA signals among other subcompartments. One limitation of this study is the use of only a lymphoblastoid cell line Hi‐C data in deriving the subcompartments information. As cfDNA also originates from other cell lines, there might be other interpretations to our observations when Hi‐C data from other cell lines in the hematopoietic system are available. For example, the age‐associated change of signals in subcompartments might arise from the change in the composition of other cell types that contribute to the cfDNA, such as those of erythroid origins, which could have a different subcompartment organization. Nevertheless, the changes that we observed are consistent with previous studies that demonstrated a reduction in the peripheral heterochromatic compartment and an overall compaction of euchromatin with senescence (De Cecco, Criscione, Peckham, et al., 2013; De Cecco, Criscione, Peterson, et al., 2013). Furthermore, we observed depleted signals near TSS and TTS especially in unhealthy centenarians. Such change in the nucleosome landscape might dictate the chromatin changes in aging cells and suggest a crucial hypothesis on aging and aging‐related diseases as a continuum during lifespan trajectories (Franceschi et al., 2018).

The activation of L1HS retrotransposon has also been implicated in aging (De Cecco, Criscione, Peckham, et al., 2013; De Cecco, Criscione, Peterson, et al., 2013). Unlike genes, L1HS has an internal promoter at the 5′UTR (Speek, 2001). We detected a relative loss of cfDNA signals in these locations with age, with unhealthy centenarian and old group showing the lowest cfDNA coverage compared to young individuals. We propose that cfDNA can be used as a method to identify L1HS activation in vivo. AluY also showed a similar trend of cfDNA level changes with age as L1HS, suggesting a derepression of this repetitive element in aging and cancer.

We also noticed that the fraction of reads mapping to the mitochondrial genome is slightly increased with age although not significant (data not shown). The key limitation that restricts our ability to investigate this feature is the short‐length cell‐free mtDNA (cf‐mtDNA) (Zhang, Nakahira, Guo, Choi, & Gu, 2016) and the protocol that we used to extract cfDNA from the plasma does not specifically enrich for short DNA. Therefore, the signals from our samples might not represent the whole cf‐mtDNA population in the plasma.

Cell‐free DNA has been recently used to identify the tissue of origin of apoptotic cells (Feng, Jin, & Wu, 2018; Guo et al., 2017; Snyder et al., 2016; Sun et al., 2015). We applied one of these methods, originally developed for cancer studies (Snyder et al., 2016), to our cfDNA data and observed that the magnitude of the shifts in tissues ranking with age was lower compared to what has been reported in cancer patients, suggesting a moderate‐to‐low increase in cell death with age as compared to cancer. We did not detect any changes in tissue ranking in healthy centenarian compared to young, but we observed a few tissues increased in ranking in old and unhealthy centenarians compared to young. Because the methodology we used was developed in the context of conditions displaying large shifts in cfDNA tissue composition, we cannot exclude that the age‐dependent shifts might be below its detection limit. For example, one of the limitations of this method is the low correlation between nucleosome profiles at genes and gene expression (Supporting Information Figure S6b), which reduces its sensitivity and specificity when a large number of tissue types are queried. Alternative approaches that use tissue‐specific nucleosome profiles as opposed to gene expression might improve our ability to detect more subtle changes in cell death across multiple tissues.

Consistently across our study, we noted more similarity in the cfDNA profiles, both globally (compartments) and locally (e.g., TTS and TSS), between young and healthy centenarians, as opposed to old and unhealthy centenarians. Hence, our study suggests cfDNA profiling could be used not only as a biomarker of age but also as a predictor of health status. However, due to the small number of subjects used, our power to translate these findings into a real predictor is very limited. Larger cohorts, more age groups, and health conditions would be needed to properly take into account the interindividual variability, as well as any potential association with lifestyle and nutritional differences.

## 4 EXPERIMENTAL PROCEDURES

### 4.1 Volunteer recruitment

Twelve volunteers across three different age groups; that is, three healthy 25‐year‐old ± 0.5 (*SD*) young subjects, three healthy 71‐year‐old ± 1.6 elderly subjects, and six 101.8‐year‐old ± 1.1 centenarians were recruited. All volunteers were enrolled in Bologna, Italy, and centenarians comprised two extreme phenotypes, that is, three healthy and three unhealthy subjects. Ethical Committee of Sant'Orsola‐Malpighi Hospital (Bologna, Italy) approved the protocol (EM/26/2014/U with reference to 22/2007/U/Tess). Centenarians’ phenotype was defined by a specific questionnaire related to lifestyle, and physical and cognitive status. Healthy centenarians were characterized as having good cognitive performance, that is, SMMSE>24 scores, and ability to walk with high ADL (Activities of Daily Living) score. In contrast, unhealthy centenarians were demented, not able to perform the SMMSE and bedridden. In all groups, the F:M ratio was 2:1, except for unhealthy centenarians that consisted of all females.

### 4.2 Sample processing

Cell‐free DNA was isolated from 3 to 4 ml of plasma using the QIAamp Circulating Nucleic Acid Kit (Qiagen, Hilden, Germany). At least 20 ng of total DNA from each sample was extracted and quantified using Qubit fluorometer (Thermo Fisher Scientific, Waltham, MA, USA).

### 4.3 Sequencing library preparation and sequencing

Sequencing libraries were prepared from the purified DNA using Ovation^®^ Ultralow Library Systems V2 library preparation kit (NuGen Technologies, San Carlos, CA, USA). Sequencing was performed on HiseqX‐Ten Illumina platform at BGI Beijing Genomics Institute, Hong Kong.

### 4.4 cfDNA sequencing reads alignment and processing

Paired‐end reads were aligned to hs37d5 genome using BWA‐MEM 0.7.12 (Heng Li, 2013). Reads mapping to the mitochondrial, X and Y chromosomes, and reads with MAPQ 0 were removed. Duplicate reads were discarded using picard‐tools‐1.88. Reads that were soft‐clipped of <75 bp were retained. DANPOS2 (Chen et al., 2013) was used to assess the cfDNA signals at TSS, TTS, and CTCF. BAM files of triplicates from each age group were merged using SAMTools (Li et al., 2009) and used to identify fragment sizes using picard‐tools‐1.88 (http://broadinstitute.github.io/picard). Subcompartments information was obtained from the Hi‐C data of GM12878 (Rao et al., 2014). cfDNA signals across subcompartments were identified by the number of reads mapping to each 100‐kb region after downsampling all samples to 46,301,323 reads. The reads were averaged between replicates to generate the average cfDNA signals. To reconstruct the 3D organization of chr 11 at 100 kb resolution, Hi‐C matrix of GM12878 was obtained using juicer (Durand et al., 2016) and the xyz coordinates were obtained using ShRec3D (Lesne, Riposo, Roger, Cournac, & Mozziconacci, 2014).

### 4.5 Genome browser view

GM12878 MNase‐seq from ENCODE (GEO: GSM920558) data were visualized in UCSC Genome Browser, alongside the cfDNA signals.

### 4.6 Changes of cfDNA signals in subcompartments

DESeq2 1.20.0 (Love, Huber, & Anders, 2014) was used to identify the log fold change of each 100 kb region between age groups. MCMCglmm (Hadfield, 2010) with default parameters was used to identify significant changes of cfDNA signals in different subcompartments. Age group was used as the fixed factor predictors, and the 100‐kb binning regions in each subcompartment were set as the random factor.

### 4.7 Tissues RNA‐Seq

Gene expression values (RPKM) from different human tissues of different ages were obtained from the GTEx portal. First, we filtered by data that were prepared using TrueSeq.v1 and retained only samples that are <25 years old or>65 years old. Second, we removed samples that were reported with disease and overdose. Third, we excluded sex‐associated tissues: ovary, prostate, vagina, uterus, testis from our analysis. Fourth, we removed flagged tissues in the GTEx metadata and duplicate tissues from the same donor. Lastly, we removed tissues with <5 donors. The contributing tissues analysis was done with a total of 456 tissues from 37 donors and 33 tissue subtypes.

### 4.8 Identification of cfDNA contributing tissues

We used the method developed by Snyder et al. (2016) in identifying contributing tissues. Briefly, we computed the windows protection score (WPS) across 10‐kb downstream of TSS of each gene. We converted the oscillating signals into frequency using fast Fourier transformation method and performed Pearson's correlation between the FFT intensities and gene expression values from the GTEx portal. To identify the shift of contributing tissues ranks, we performed Kruskal–Wallis test and subsequently Dunn test for multiple pairwise comparisons between age groups. The shift in contributing tissues of cancer dataset obtained from Snyder et al was identified using the same method. *p*‐Values were adjusted using FDR.

### 4.9 CTCF, L1HS, and dimeric AluY nucleosome signals

Cell‐free DNA BAM files were used to identify average nucleosome signals at 39,362 CTCF‐binding sites, 1,371 L1HS regions, and 94,024 dimeric AluY sites. Dimeric AluY are defined as 280–320 bp in length. CTCF‐binding sites are obtained from GEO: GSM733752.

### 4.10 Cancer and healthy individuals cfDNA sequencing data processing

Cell‐free DNA sequencing data from IH02 healthy (SRR2130051), IH03 healthy (SRR2130052), and IC17 liver cancer (SRR2130016) under accession number GSE71378 (Snyder et al., 2016) were downloaded and processed the same way as our cfDNA samples to identify the tissues of origin. Prostate cancer cfDNA samples, IC26 (SRR2130025), IC13 (SRR2130012), IC40 (SRR2130038), a healthy sample, IH02 (SRR2130051), and a liver cancer sample, IC17 (SRR2130016) under accession number GSE71378 were downloaded and processed the same way as our cfDNA samples to identify the signals at L1HS and AluY.

### 4.11 GM12878 MNase‐seq data processing

GM12878 MNase‐seq data deposited under accession number GSM920558 were downloaded and aligned to hs37d5 using bowtie (Langmead, Trapnell, Pop, & Salzberg, 2009). Mitochondrial, X and Y chromosomes mapped reads were removed. Duplicate reads were discarded using picard‐tools‐1.88, and 100‐kb MNase‐seq signals across subcompartments were identified by the number of reads mapping to each 100‐kb region.

### 4.12 GM12878 ATAC‐seq data processing

GM12878 ATAC‐seq data deposited under accession number GSE47753 were downloaded and aligned to hs37d5 using BWA‐MEM 0.7.12 (Li, 2013). Duplicates were removed, and peaks were called using MACS 2.1.1 (Zhang et al., 2008). The number of reads in each 100 kb of the subcompartments was identified.

### 4.13 cfDNA coverage at repetitive elements

Paired‐end reads were aligned to hs37d5 using bowtie2 (Langmead & Salzberg, 2012) and separated into uniquely mapped and multi‐mapped reads. Uniquely mapped reads were counted at each repetitive element's location, and their genomic positions in hs37d5 were converted to the positions in the consensus. The counts were then averaged at each position in the consensus. Multi‐mapped reads were aligned to the repeat's assemblies representing each repetitive element subfamilies. Reads that mapped to AluY or L1HS were extracted and subsequently mapped to the respective repetitive element's consensus sequence. The read was divided by the number of repetitive element subfamilies that it mapped to, and the compiled counts in each consensus location were averaged. The final counts were defined as the sum of counts obtained from the uniquely mapped counts and that of the multi‐mapped counts and were normalized by the library size.

### 4.14 Hemato‐biochemical parameters

Centenarians were also analyzed for 32 hemato‐biochemical parameters as follows: white blood cell (WBC), red blood cell count (RBC), hemoglobin (HGB), hematocrit (HCT), mean cell volume (MCV), mean cell hemoglobin (MCH), mean cell hemoglobin concentration (MCHC), platelet (PLT), red blood cell distribution width—standard deviation (RDW‐*SD*), red blood cell distribution width—coefficient of variation (RDW‐CV), platelet distribution width (PDW), mean platelet volume (MPV), platelet large cell ratio (PLCR), neutrophil, lymphocyte, monocyte, eosinophil, basophil, glycemia, uric acid, creatinine, total protein, total cholesterol, high‐density lipoprotein (HDL), low‐density lipoprotein (LDL), triglycerides, glutamate‐pyruvate transaminase (GPT), albumin (ALB), sodium (NA), potassium (K), C‐reactive protein (CRP), and iron.

### 4.15 Data availability

Cell‐free DNA sequenced reads have been deposited in the NCBI GEO database with accession number GSE114511.

## CONFLICT OF INTERESTS

None declared.

## AUTHOR CONTRIBUTIONS

NN, CF, MC, and AMCF conceived and designed the study; MC, CM, and GP collected samples and extracted the cell‐free DNA; YVT and NN designed the data analysis; YVT performed the data analysis; YVT, NN, MC, and CF wrote the manuscript.

## References

[acel12890-bib-0001] Anwar, S. L. , Wulaningsih, W. , & Lehmann, U. (2017). Transposable elements in human cancer: Causes and consequences of deregulation. International Journal of Molecular Sciences, 18(5), 974 10.3390/ijms18050974 PMC5454887 28471386

[acel12890-bib-0002] Arosio, B. , Ostan, R. , Mari, D. , Damanti, S. , Ronchetti, F. , Arcudi, S. , … Monti, D. (2017). Cognitive status in the oldest old and centenarians: A condition crucial for quality of life methodologically difficult to assess. Mechanisms of Ageing and Development, 165(Pt B), 185–194. 10.1016/j.mad.2017.02.010 28286214

[acel12890-bib-0003] Capri, M. , Moreno‐Villanueva, M. , Cevenini, E. , Pini, E. , Scurti, M. , Borelli, V. , … Franceschi, C. (2015). MARK‐AGE population: From the human model to new insights. Mechanisms of Ageing and Development, 151, 13–17. 10.1016/j.mad.2015.03.010 25843237

[acel12890-bib-0004] Castagne, R. , Gares, V. , Karimi, M. , Chadeau‐Hyam, M. , Vineis, P. , Delpierre, C. , … Lifepath, C. (2018). Allostatic load and subsequent all‐cause mortality: Which biological markers drive the relationship? Findings from a UK birth cohort. European Journal of Epidemiology, 33(5), 441–458. 10.1007/s10654-018-0364-1 29476357 PMC5968064

[acel12890-bib-0005] Chen, B. H. , Marioni, R. E. , Colicino, E. , Peters, M. J. , Ward‐Caviness, C. K. , Tsai, P. C. , … Horvath, S. (2016). DNA methylation‐based measures of biological age: Meta‐analysis predicting time to death. Aging, 8(9), 1844–1865. 10.18632/aging.101020 27690265 PMC5076441

[acel12890-bib-0006] Chen, H. , Tian, Y. , Shu, W. , Bo, X. , & Wang, S. (2012). Comprehensive identification and annotation of cell type‐specific and ubiquitous CTCF‐binding sites in the human genome. PLoS One, 7(7), e41374 10.1371/journal.pone.0041374 22829947 PMC3400636

[acel12890-bib-0007] Chen, K. , Xi, Y. , Pan, X. , Li, Z. , Kaestner, K. , Tyler, J. , … Li, W. (2013). DANPOS: Dynamic analysis of nucleosome position and occupancy by sequencing. Genome Research, 23(2), 341–351. 10.1101/gr.142067.112 23193179 PMC3561875

[acel12890-bib-0008] Ciccocioppo, R. , Di Sabatino, A. , Luinetti, O. , Rossi, M. , Cifone, M. G. , & Corazza, G. R. (2002). Small bowel enterocyte apoptosis and proliferation are increased in the elderly. Gerontology, 48(4), 204–208. 10.1159/000058351 12053108

[acel12890-bib-0009] Consortium, E. P (2012). An integrated encyclopedia of DNA elements in the human genome. Nature, 489(7414), 57–74. 10.1038/nature11247 22955616 PMC3439153

[acel12890-bib-0010] Criscione, S. W. , Teo, Y. V. , & Neretti, N. (2016). The chromatin landscape of cellular senescence. Trends in Genetics, 32(11), 751–761. 10.1016/j.tig.2016.09.005 27692431 PMC5235059

[acel12890-bib-0011] Criscione, S. W. , Zhang, Y. , Thompson, W. , Sedivy, J. M. , & Neretti, N. (2014). Transcriptional landscape of repetitive elements in normal and cancer human cells. BMC Genomics, 15, 583 10.1186/1471-2164-15-583 25012247 PMC4122776

[acel12890-bib-0012] De Cecco, M. , Criscione, S. W. , Peterson, A. L. , Neretti, N. , Sedivy, J. M. , & Kreiling, J. A. (2013). Transposable elements become active and mobile in the genomes of aging mammalian somatic tissues. Aging, 5(12), 867–883. 10.18632/aging.100621 24323947 PMC3883704

[acel12890-bib-0013] De Cecco, M. , Criscione, S. W. , Peckham, E. J. , Hillenmeyer, S. , Hamm, E. A. , Manivannan, J. , … Sedivy, J. M. (2013). Genomes of replicatively senescent cells undergo global epigenetic changes leading to gene silencing and activation of transposable elements. Aging Cell, 12(2), 247–256. 10.1111/acel.12047 23360310 PMC3618682

[acel12890-bib-0014] De Vlaminck, I. , Martin, L. , Kertesz, M. , Patel, K. , Kowarsky, M. , Strehl, C. , … Quake, S. R. (2015). Noninvasive monitoring of infection and rejection after lung transplantation. Proceedings of the National Academy of Sciences USA, 112(43), 13336–13341. 10.1073/pnas.1517494112 PMC4629384 26460048

[acel12890-bib-0015] Durand, N. C. , Shamim, M. S. , Machol, I. , Rao, S. S. , Huntley, M. H. , Lander, E. S. , & Aiden, E. L. (2016). Juicer provides a one‐click system for analyzing loop‐resolution Hi‐C experiments. Cell Systems, 3(1), 95–98. 10.1016/j.cels.2016.07.002 27467249 PMC5846465

[acel12890-bib-0016] Feng, H. , Jin, P. , & Wu, H. (2018). Disease prediction by cell‐free DNA methylation. Briefings in Bioinformatics, 10.1093/bib/bby029 PMC6556903 29672679

[acel12890-bib-0017] Franceschi, C. , & Campisi, J. (2014). Chronic inflammation (inflammaging) and its potential contribution to age‐associated diseases. Journals of Gerontology. Series A, Biological Sciences and Medical Sciences, 69(Suppl 1), S4–9. 10.1093/gerona/glu057 24833586

[acel12890-bib-0018] Franceschi, C. , Garagnani, P. , Vitale, G. , Capri, M. , & Salvioli, S. (2017). Inflammaging and 'Garb‐aging'. Trends in Endocrinology and Metabolism, 28(3), 199–212. 10.1016/j.tem.2016.09.005 27789101

[acel12890-bib-0019] Franceschi, C. , Salvioli, S. , Garagnani, P. , de Eguileor, M. , Monti, D. , & Capri, M. (2017). Immunobiography and the heterogeneity of immune responses in the elderly: A focus on inflammaging and trained immunity. Frontiers in Immunology, 8, 982 10.3389/fimmu.2017.00982 28861086 PMC5559470

[acel12890-bib-0020] Franceschi, C. , Garagnani, P. , Morsiani, C. , Conte, M. , Santoro, A. , Grignolio, A. , … Salvioli, S. (2018). The continuum of aging and age‐related diseases: Common mechanisms but different rates. Frontiers in Medicine, 5, 61 10.3389/fmed.2018.00061 29662881 PMC5890129

[acel12890-bib-0021] Fu, Y. , Sinha, M. , Peterson, C. L. , & Weng, Z. (2008). The insulator binding protein CTCF positions 20 nucleosomes around its binding sites across the human genome. PLoS Genetics, 4(7), e1000138 10.1371/journal.pgen.1000138 18654629 PMC2453330

[acel12890-bib-0022] Guo, S. , Diep, D. , Plongthongkum, N. , Fung, H. L. , Zhang, K. , & Zhang, K. (2017). Identification of methylation haplotype blocks aids in deconvolution of heterogeneous tissue samples and tumor tissue‐of‐origin mapping from plasma DNA. Nature Genetics, 49(4), 635–642. 10.1038/ng.3805 28263317 PMC5374016

[acel12890-bib-0023] Hadfield, J. D. (2010). MCMC methods for multi‐response generalized linear mixed models: The MCMCglmm R package. Journal of Statistical Software, 33(2), 1–22.20808728

[acel12890-bib-0024] Horvath, S. (2013). DNA methylation age of human tissues and cell types. Genome Biology, 14(10), R115 10.1186/gb-2013-14-10-r115 24138928 PMC4015143

[acel12890-bib-0025] Horvath, S. , Garagnani, P. , Bacalini, M. G. , Pirazzini, C. , Salvioli, S. , Gentilini, D. , … Franceschi, C. (2015). Accelerated epigenetic aging in Down syndrome. Aging Cell, 14(3), 491–495. 10.1111/acel.12325 25678027 PMC4406678

[acel12890-bib-0026] Horvath, S. , Pirazzini, C. , Bacalini, M. G. , Gentilini, D. , Di Blasio, A. M. , Delledonne, M. , … Franceschi, C. (2015). Decreased epigenetic age of PBMCs from Italian semi‐supercentenarians and their offspring. Aging, 7(12), 1159–1170. 10.18632/aging.100861 26678252 PMC4712339

[acel12890-bib-0027] Hu, Z. , Chen, K. , Xia, Z. , Chavez, M. , Pal, S. , Seol, J. H. , … Tyler, J. K. (2014). Nucleosome loss leads to global transcriptional up‐regulation and genomic instability during yeast aging. Genes & Development, 28(4), 396–408. 10.1101/gad.233221.113 24532716 PMC3937517

[acel12890-bib-0029] Ivanov, M. , Baranova, A. , Butler, T. , Spellman, P. , & Mileyko, V. (2015). Non‐random fragmentation patterns in circulating cell‐free DNA reflect epigenetic regulation. BMC Genomics, 16(Suppl 13), S1 10.1186/1471-2164-16-S13-S1 PMC4686799 26693644

[acel12890-bib-0030] Jung, K. , Fleischhacker, M. , & Rabien, A. (2010). Cell‐free DNA in the blood as a solid tumor biomarker – A critical appraisal of the literature. Clinica Chimica Acta, 411(21–22), 1611–1624. 10.1016/j.cca.2010.07.032 20688053

[acel12890-bib-0031] Jylhava, J. , Kotipelto, T. , Raitala, A. , Jylha, M. , Hervonen, A. , & Hurme, M. (2011). Aging is associated with quantitative and qualitative changes in circulating cell‐free DNA: The vitality 90+ study. Mechanisms of Ageing and Development, 132(1–2), 20–26. 10.1016/j.mad.2010.11.001 21078336

[acel12890-bib-0032] Jylhava, J. , Nevalainen, T. , Marttila, S. , Jylha, M. , Hervonen, A. , & Hurme, M. (2013). Characterization of the role of distinct plasma cell‐free DNA species in age‐associated inflammation and frailty. Aging Cell, 12(3), 388–397. 10.1111/acel.12058 23438186

[acel12890-bib-0033] Ke, W. L. , Zhao, W. H. , & Wang, X. Y. (2015). Detection of fetal cell‐free DNA in maternal plasma for Down syndrome, Edward syndrome and Patau syndrome of high risk fetus. International Journal of Clinical and Experimental Medicine, 8(6), 9525–9530. 26309618 PMC4538030

[acel12890-bib-0034] Kooman, J. P. , Kotanko, P. , Schols, A. M. , Shiels, P. G. , & Stenvinkel, P. (2014). Chronic kidney disease and premature ageing. Nature Reviews Nephrology, 10(12), 732–742. 10.1038/nrneph.2014.185 25287433

[acel12890-bib-0035] Kooman, J. P. , Dekker, M. J. , Usvyat, L. A. , Kotanko, P. , van der Sande, F. M. , Schalkwijk, C. G. , … Stenvinkel, P. (2017). Inflammation and premature aging in advanced chronic kidney disease. American Journal of Physiology. Renal Physiology, 313(4), F938–F950. 10.1152/ajprenal.00256.2017 28701312

[acel12890-bib-0036] Langmead, B. , & Salzberg, S. L. (2012). Fast gapped‐read alignment with Bowtie 2. Nature Methods, 9(4), 357–359. 10.1038/nmeth.1923 22388286 PMC3322381

[acel12890-bib-0037] Langmead, B. , Trapnell, C. , Pop, M. , & Salzberg, S. L. (2009). Ultrafast and memory‐efficient alignment of short DNA sequences to the human genome. Genome Biology, 10(3), R25 10.1186/gb-2009-10-3-r25 19261174 PMC2690996

[acel12890-bib-0038] Lehmann‐Werman, R. , Neiman, D. , Zemmour, H. , Moss, J. , Magenheim, J. , Vaknin‐Dembinsky, A. , … Dor, Y. (2016). Identification of tissue‐specific cell death using methylation patterns of circulating DNA. Proceedings of the National Academy of Sciences USA, 113(13), E1826–1834. 10.1073/pnas.1519286113 PMC4822610 26976580

[acel12890-bib-0039] Lesne, A. , Riposo, J. , Roger, P. , Cournac, A. , & Mozziconacci, J. (2014). 3D genome reconstruction from chromosomal contacts. Nature Methods, 11(11), 1141–1143. 10.1038/nmeth.3104 25240436

[acel12890-bib-0040] Li, G. , & Zhou, L. (2013). Genome‐wide identification of chromatin transitional regions reveals diverse mechanisms defining the boundary of facultative heterochromatin. PLoS One, 8(6), e67156 10.1371/journal.pone.0067156 23840609 PMC3696093

[acel12890-bib-0041] Li, H. (2013). Aligning sequence reads, clone sequences and assembly contigs with BWA‐MEM. ArXiv e‐prints, 1303. Retrieved from http://adsabs.harvard.edu/abs/2013arXiv1303.3997L

[acel12890-bib-0042] Li, H. , Handsaker, B. , Wysoker, A. , Fennell, T. , Ruan, J. , Homer, N. , … Genome Project Data Processing, S (2009). The sequence alignment/Map format and SAMtools. Bioinformatics, 25(16), 2078–2079. 10.1093/bioinformatics/btp352 19505943 PMC2723002

[acel12890-bib-0043] Liu, X. , Li, P. , Widlak, P. , Zou, H. , Luo, X. , Garrard, W. T. , & Wang, X. (1998). The 40‐kDa subunit of DNA fragmentation factor induces DNA fragmentation and chromatin condensation during apoptosis. Proceedings of the National Academy of Sciences USA, 95(15), 8461–8466. 10.1073/pnas.95.15.8461 PMC21098 9671700

[acel12890-bib-0044] Lo, Y. M. , Chan, K. C. , Sun, H. , Chen, E. Z. , Jiang, P. , Lun, F. M. , … Chiu, R. W. (2010). Maternal plasma DNA sequencing reveals the genome‐wide genetic and mutational profile of the fetus. Science Translational Medicine, 2(61), 61ra91 10.1126/scitranslmed.3001720 21148127

[acel12890-bib-0045] Love, M. I. , Huber, W. , & Anders, S. (2014). Moderated estimation of fold change and dispersion for RNA‐seq data with DESeq2. Genome Biology, 15(12), 550 10.1186/s13059-014-0550-8 25516281 PMC4302049

[acel12890-bib-0046] Mieczkowski, J. , Cook, A. , Bowman, S. K. , Mueller, B. , Alver, B. H. , Kundu, S. , … Tolstorukov, M. Y. (2016). MNase titration reveals differences between nucleosome occupancy and chromatin accessibility. Nature Communications, 7, 11485 10.1038/ncomms11485 PMC4859066 27151365

[acel12890-bib-0047] Miura, Y. , & Endo, T. (2016). Glycomics and glycoproteomics focused on aging and age‐related diseases – Glycans as a potential biomarker for physiological alterations. Biochimica et Biophysica Acta, 1860(8), 1608–1614. 10.1016/j.bbagen.2016.01.013 26801879

[acel12890-bib-0048] Naumann, T. A. , & Reznikoff, W. S. (2002). Tn5 transposase active site mutants. Journal of Biological Chemistry, 277(20), 17623–17629. 10.1074/jbc.M200742200 11877443

[acel12890-bib-0049] Nishimoto, S. , Fukuda, D. , Higashikuni, Y. , Tanaka, K. , Hirata, Y. , Murata, C. , … Sata, M. (2016). Obesity‐induced DNA released from adipocytes stimulates chronic adipose tissue inflammation and insulin resistance. Science Advances, 2(3), e1501332 10.1126/sciadv.1501332 27051864 PMC4820373

[acel12890-bib-0050] Ong, C. T. , & Corces, V. G. (2014). CTCF: An architectural protein bridging genome topology and function. Nature Reviews Genetics, 15(4), 234–246. 10.1038/nrg3663 PMC4610363 24614316

[acel12890-bib-0051] Ostan, R. , Monti, D. , Mari, D. , Arosio, B. , Gentilini, D. , Ferri, E. , … Vitale, G. (2018). Heterogeneity of thyroid function and impact of peripheral thyroxine deiodination in centenarians and semi‐supercentenarians: Association with functional status and mortality. Journals of Gerontology. Series A, Biological Sciences and Medical Sciences, 10.1093/gerona/gly194 30165411

[acel12890-bib-0052] Pinti, M. , Cevenini, E. , Nasi, M. , De Biasi, S. , Salvioli, S. , Monti, D. , … Cossarizza, A. (2014). Circulating mitochondrial DNA increases with age and is a familiar trait: Implications for “inflamm‐aging”. European Journal of Immunology, 44(5), 1552–1562. 10.1002/eji.201343921 24470107

[acel12890-bib-0053] Pollack, M. , Phaneuf, S. , Dirks, A. , & Leeuwenburgh, C. (2002). The role of apoptosis in the normal aging brain, skeletal muscle, and heart. Annals of the New York Academy of Sciences, 959, 93–107. 10.1111/j.1749-6632.2002.tb02086.x 11976189

[acel12890-bib-0054] Rao, S. S. , Huntley, M. H. , Durand, N. C. , Stamenova, E. K. , Bochkov, I. D. , Robinson, J. T. , … Aiden, E. L. (2014). A 3D map of the human genome at kilobase resolution reveals principles of chromatin looping. Cell, 159(7), 1665–1680. 10.1016/j.cell.2014.11.021 25497547 PMC5635824

[acel12890-bib-0055] Schones, D. E. , Cui, K. , Cuddapah, S. , Roh, T. Y. , Barski, A. , Wang, Z. , … Zhao, K. (2008). Dynamic regulation of nucleosome positioning in the human genome. Cell, 132(5), 887–898. 10.1016/j.cell.2008.02.022 18329373 PMC10894452

[acel12890-bib-0056] Snyder, M. W. , Kircher, M. , Hill, A. J. , Daza, R. M. , & Shendure, J. (2016). Cell‐free DNA comprises an in vivo nucleosome footprint that informs its tissues‐of‐origin. Cell, 164(1–2), 57–68. 10.1016/j.cell.2015.11.050 26771485 PMC4715266

[acel12890-bib-0057] Speek, M. (2001). Antisense promoter of human L1 retrotransposon drives transcription of adjacent cellular genes. Molecular and Cellular Biology, 21(6), 1973–1985. 10.1128/MCB.21.6.1973-1985.2001 11238933 PMC86790

[acel12890-bib-0058] Stroun, M. , Anker, P. , Lyautey, J. , Lederrey, C. , & Maurice, P. A. (1987). Isolation and characterization of DNA from the plasma of cancer patients. European Journal of Cancer and Clinical Oncology, 23(6), 707–712. 10.1016/0277-5379(87)90266-5 3653190

[acel12890-bib-0059] Sun, K. , Jiang, P. , Chan, K. C. , Wong, J. , Cheng, Y. K. , Liang, R. H. , … Lo, Y. M. (2015). Plasma DNA tissue mapping by genome‐wide methylation sequencing for noninvasive prenatal, cancer, and transplantation assessments. Proceedings of the National Academy of Sciences USA, 112(40), E5503–5512. 10.1073/pnas.1508736112. PMC4603482 26392541

[acel12890-bib-0060] Swergold, G. D. (1990). Identification, characterization, and cell specificity of a human LINE‐1 promoter. Molecular and Cellular Biology, 10(12), 6718–6729. 10.1128/MCB.10.12.6718 1701022 PMC362950

[acel12890-bib-0061] Tan, E. M. , Schur, P. H. , Carr, R. I. , & Kunkel, H. G. (1966). Deoxybonucleic acid (DNA) and antibodies to DNA in the serum of patients with systemic lupus erythematosus. Journal of Clinical Investigation, 45(11), 1732–1740. 10.1172/JCI105479 4959277 PMC292857

[acel12890-bib-0062] Taniuchi, H. , Anfinsen, C. B. , & Sodja, A. (1967). The amino acid sequence of an extracellular nuclease of *Staphylococcus aureus*. 3. Complete amino acid sequence. Journal of Biological Chemistry, 242(20), 4752–4758. 4294024

[acel12890-bib-0063] Tower, J. (2015). Programmed cell death in aging. Ageing Research Reviews, 23(Pt A), 90–100. 10.1016/j.arr.2015.04.002 25862945 PMC4480161

[acel12890-bib-0064] United Nations, D. o. E. a. S. A., Population Division (2017). World population prospects: The 2017 revision, key findings and advance tables. Working Paper No. ESA/P/WP/248.

[acel12890-bib-0065] Valouev, A. , Johnson, S. M. , Boyd, S. D. , Smith, C. L. , Fire, A. Z. , & Sidow, A. (2011). Determinants of nucleosome organization in primary human cells. Nature, 474(7352), 516–520. 10.1038/nature10002 21602827 PMC3212987

[acel12890-bib-0066] van der Vaart, M. , & Pretorius, P. J. (2007). The origin of circulating free DNA. Clinical Chemistry, 53(12), 2215 10.1373/clinchem.2007.092734 18267930

[acel12890-bib-0067] Vazquez‐Padron, R. I. , Lasko, D. , Li, S. , Louis, L. , Pestana, I. A. , Pang, M. , … Pham, S. M. (2004). Aging exacerbates neointimal formation, and increases proliferation and reduces susceptibility to apoptosis of vascular smooth muscle cells in mice. Journal of Vascular Surgery, 40(6), 1199–1207. 10.1016/j.jvs.2004.08.034 15622375

[acel12890-bib-0068] Zhang, R. , Nakahira, K. , Guo, X. , Choi, A. M. , & Gu, Z. (2016). Very short mitochondrial DNA fragments and heteroplasmy in human plasma. Scientific Reports, 6, 36097 10.1038/srep36097 27811968 PMC5095883

[acel12890-bib-0069] Zhang, Y. , Liu, T. , Meyer, C. A. , Eeckhoute, J. , Johnson, D. S. , Bernstein, B. E. , … Liu, X. S. (2008). Model‐based analysis of ChIP‐Seq (MACS). Genome Biology, 9(9), R137 10.1186/gb-2008-9-9-r137 18798982 PMC2592715

