# Supplementary figures and images for: Cell‐free DNA as a biomarker of aging
Source: Aging Cell. 2018 Dec 20;18(1):e12890. doi: 10.1111/acel.12890 (PMC6351822; doi:10.1111/acel.12890)

Fig S1

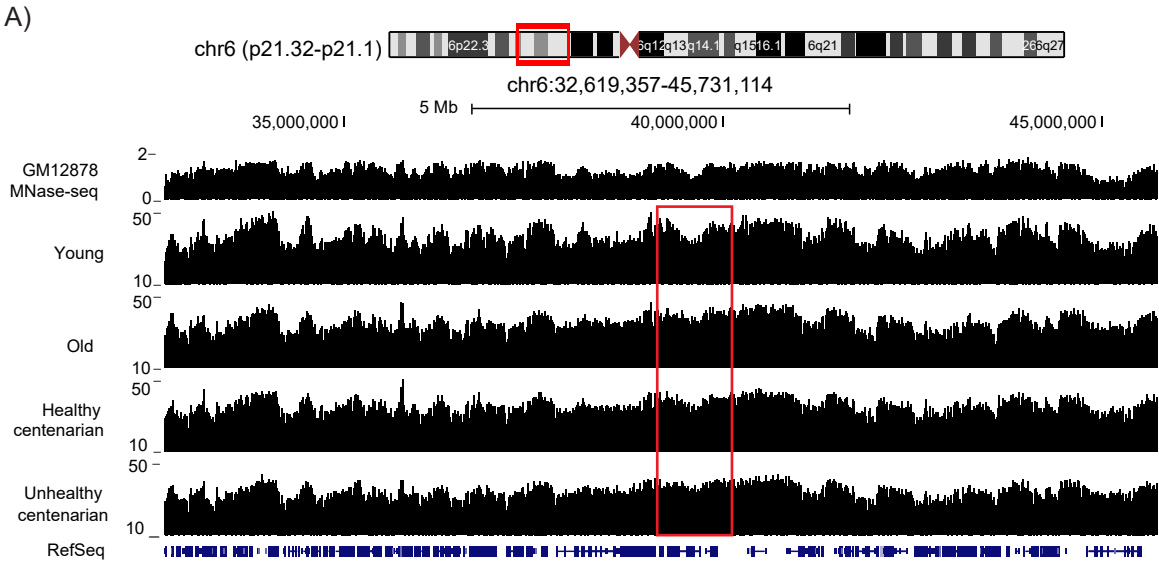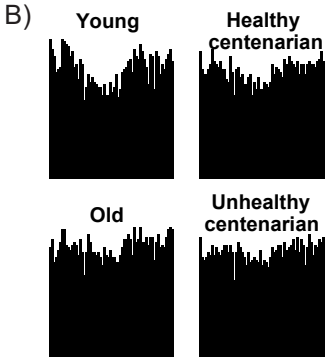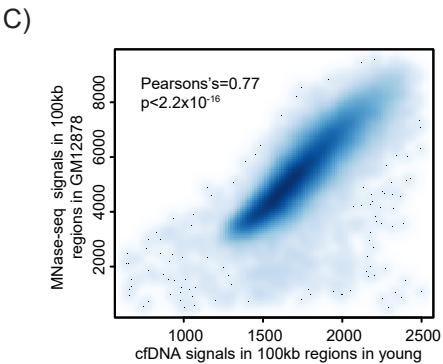

Supplement: Supplementary file 1 [file ACEL-18-e12890-s001.pdf]

Fig S2

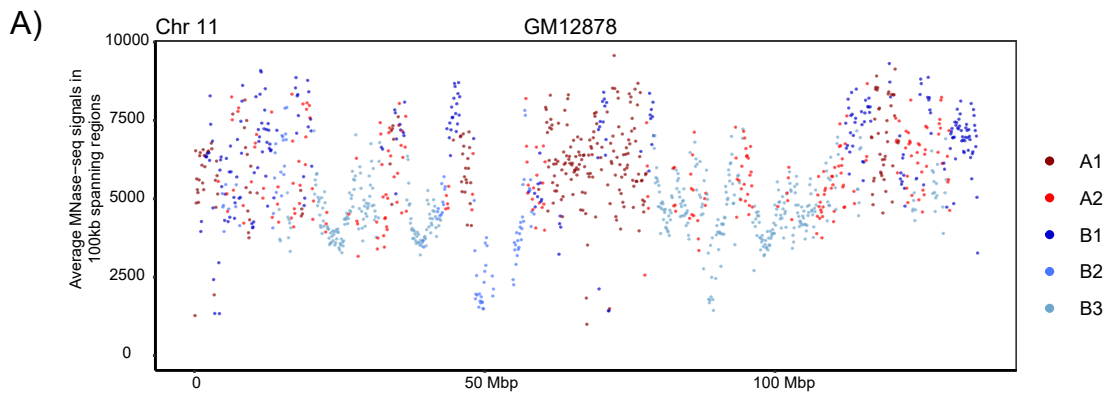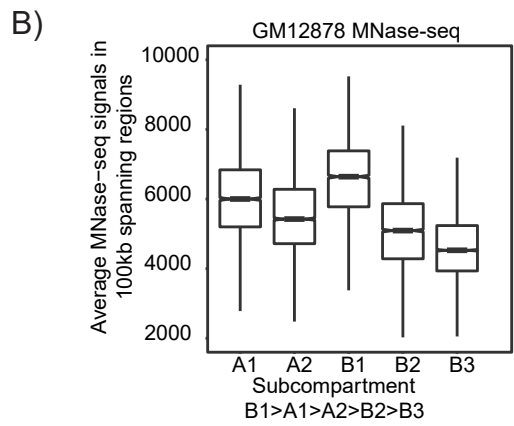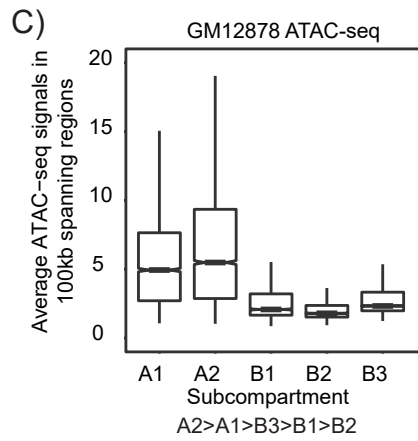

Supplement: Supplementary file 2 [file ACEL-18-e12890-s002.pdf]

Fig S3

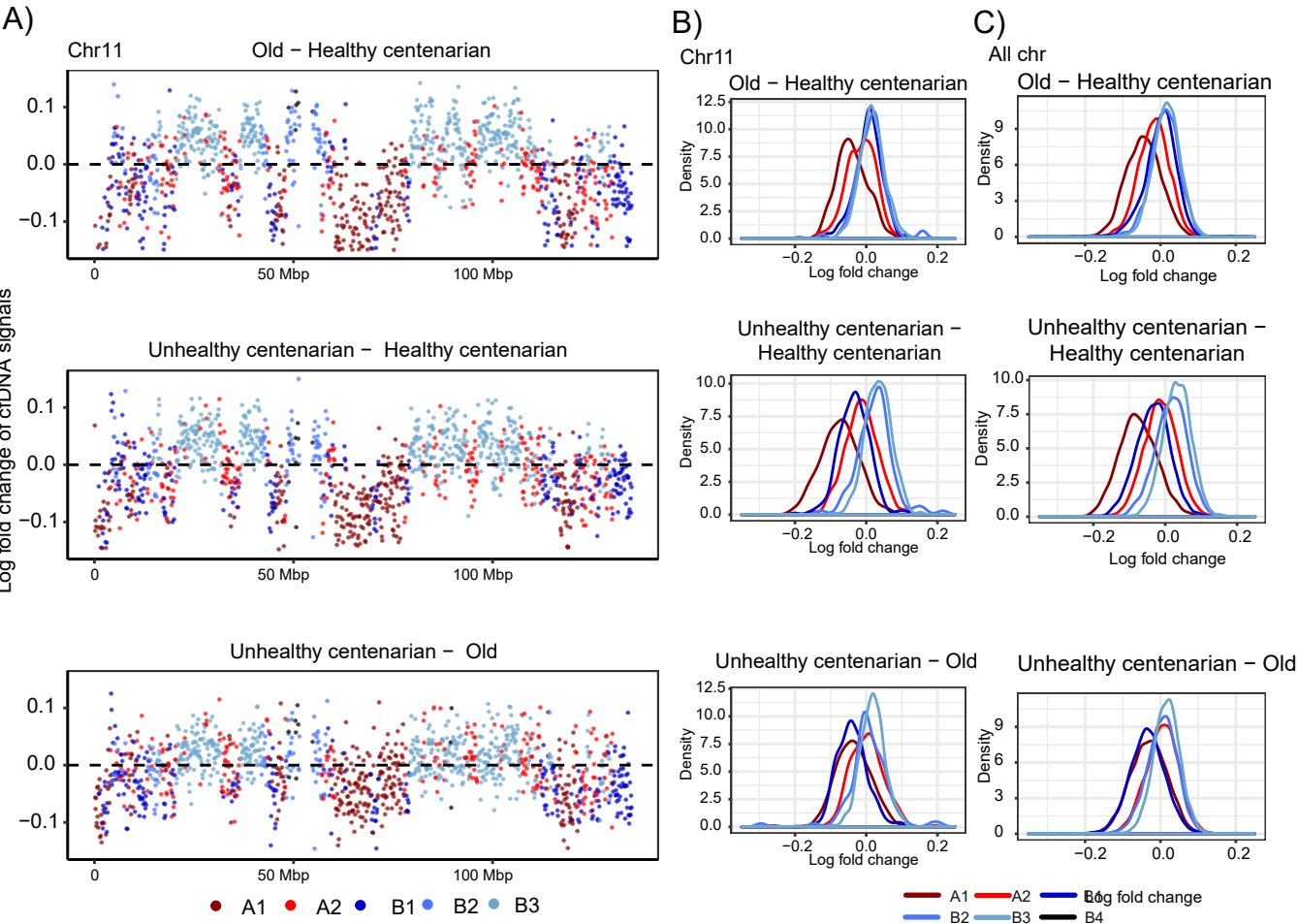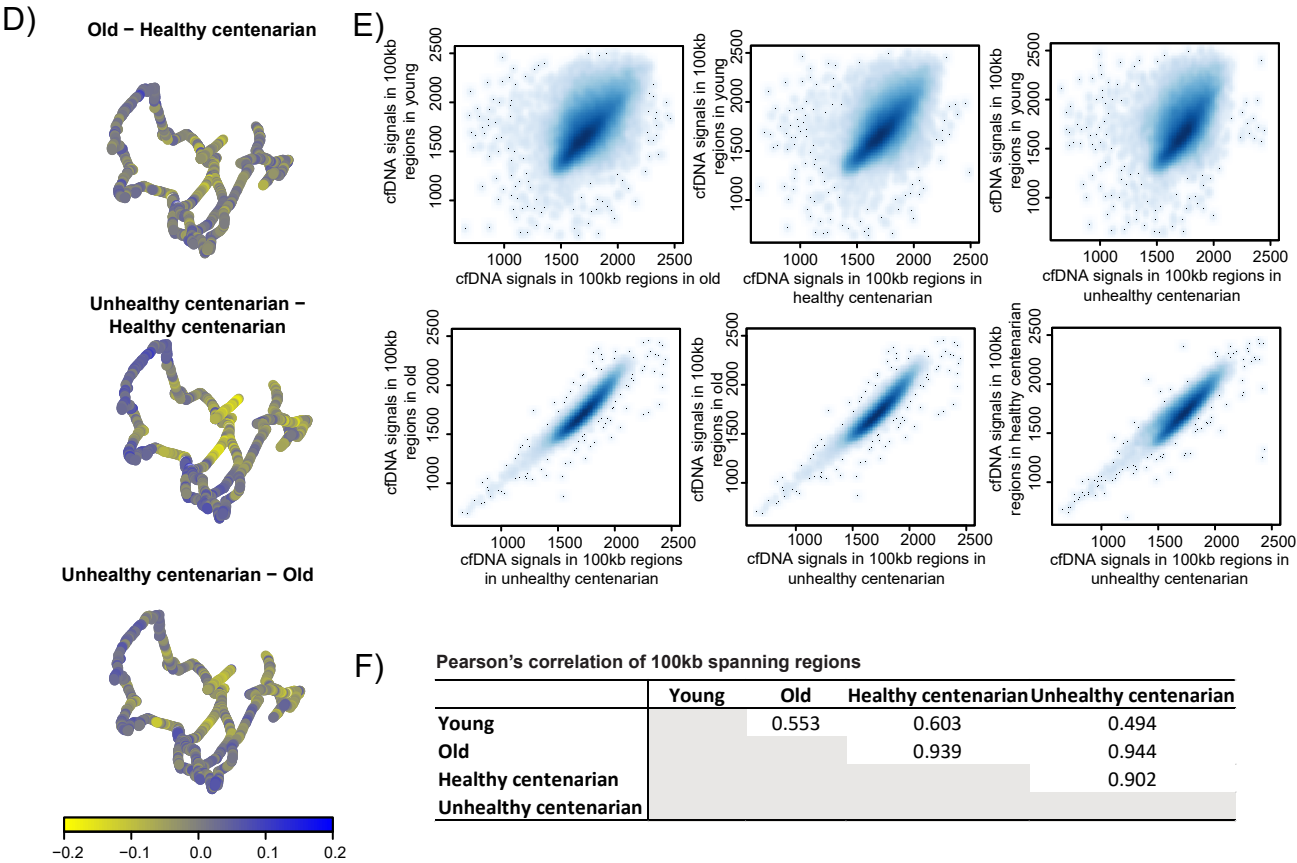

Supplement: Supplementary file 3 [file ACEL-18-e12890-s003.pdf]

Fig S4

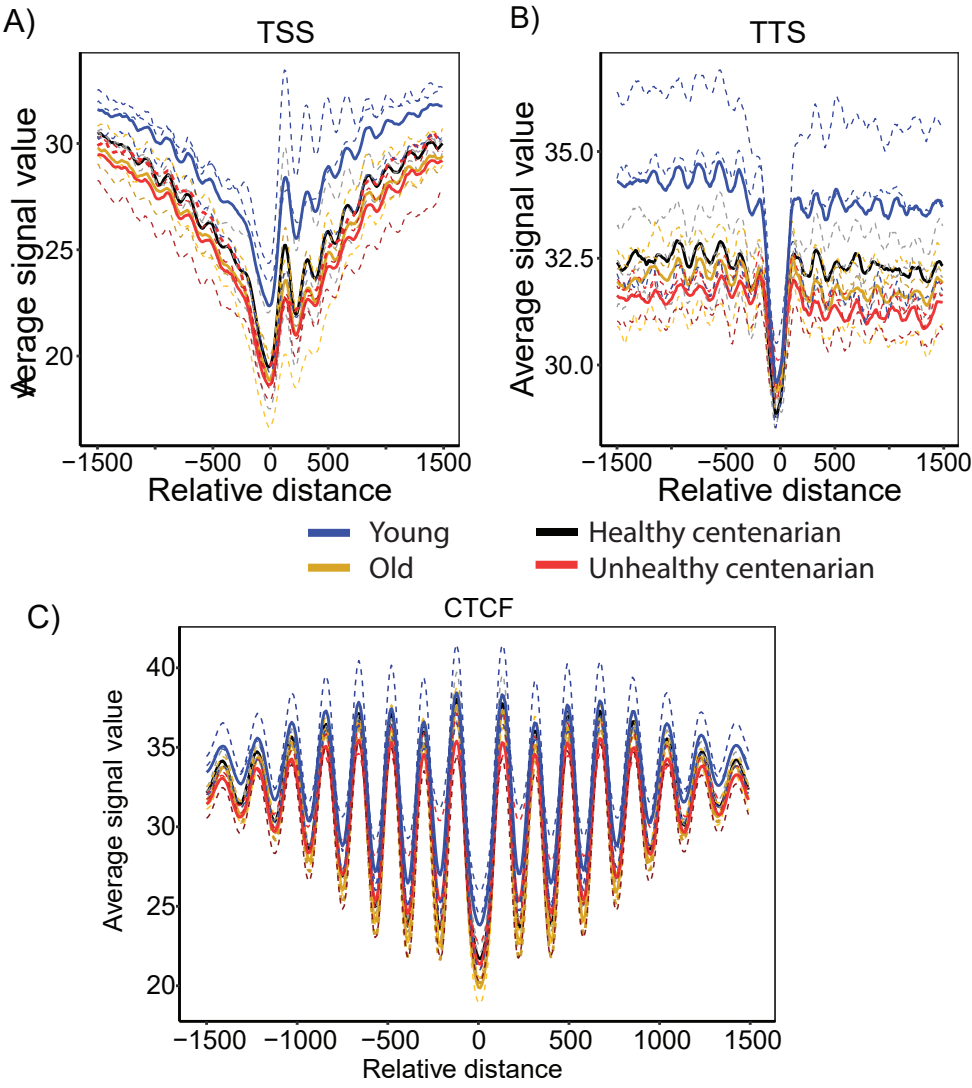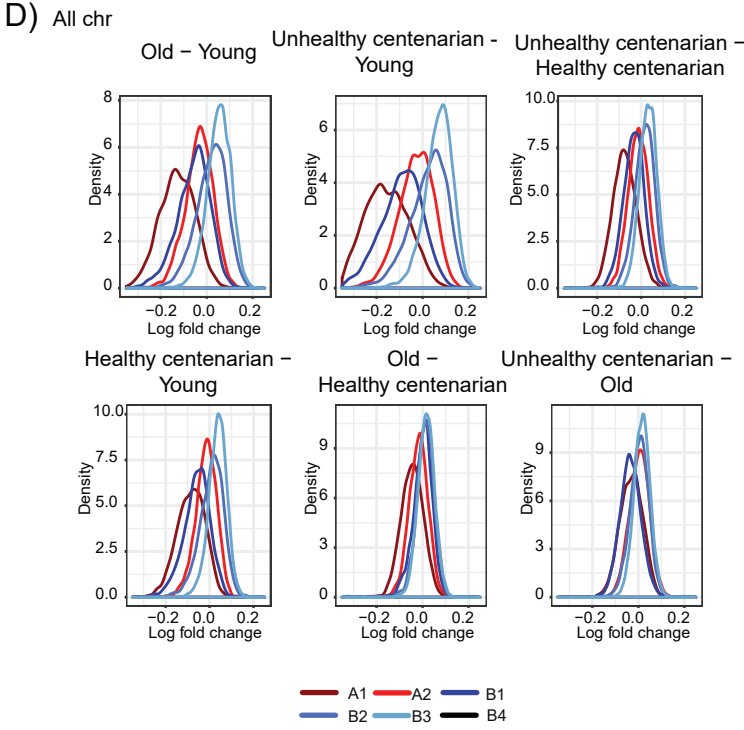

Supplement: Supplementary file 4 [file ACEL-18-e12890-s004.pdf]

Fig S5

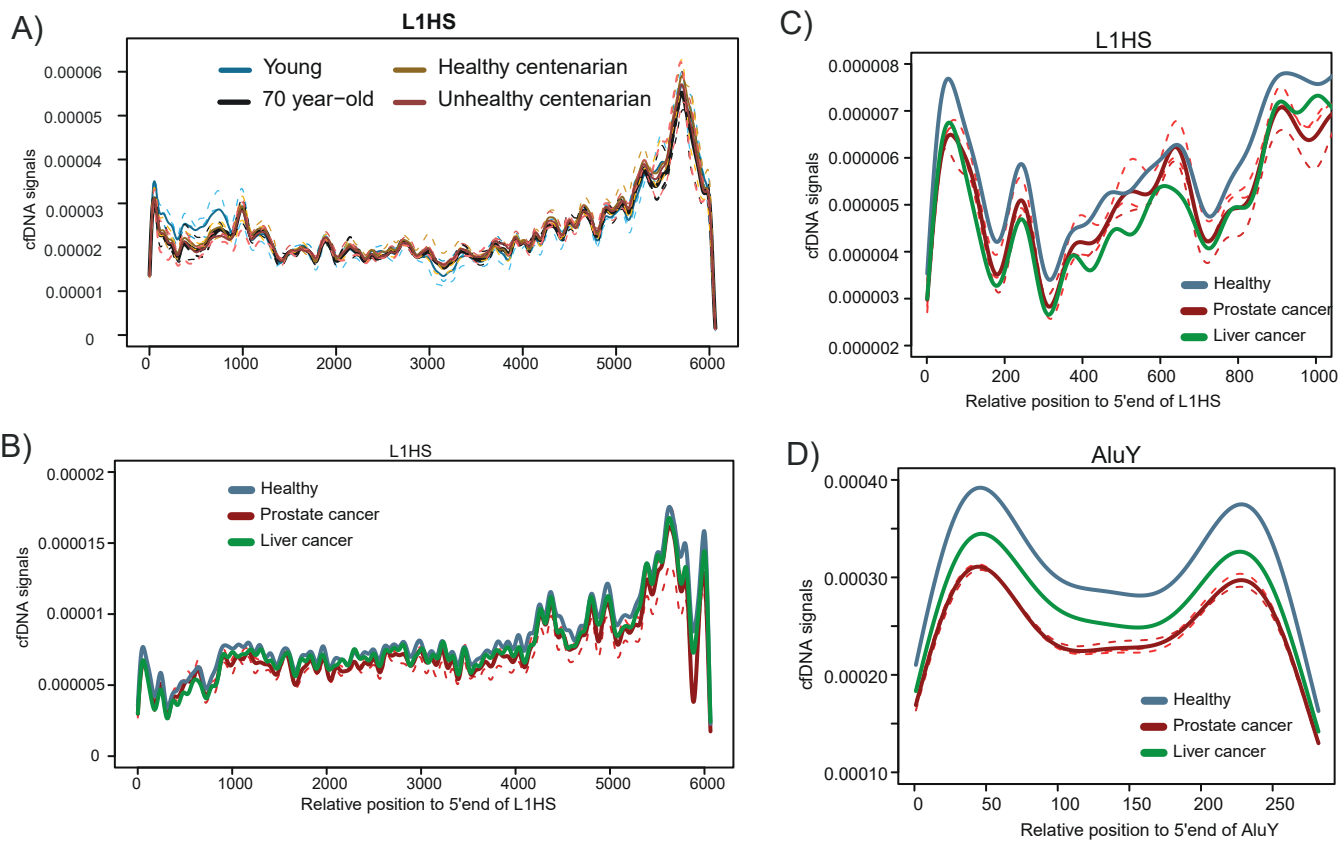

Supplement: Supplementary file 5 [file ACEL-18-e12890-s005.pdf]
