# Supplementary material for: Cell‐free DNA as a biomarker of aging
Source: Aging Cell. 2018 Dec 20;18(1):e12890. doi: 10.1111/acel.12890 (PMC6351822; doi:10.1111/acel.12890)
Supplement: Supplementary file 6 [file ACEL-18-e12890-s006.pdf]

Fig S6

A)

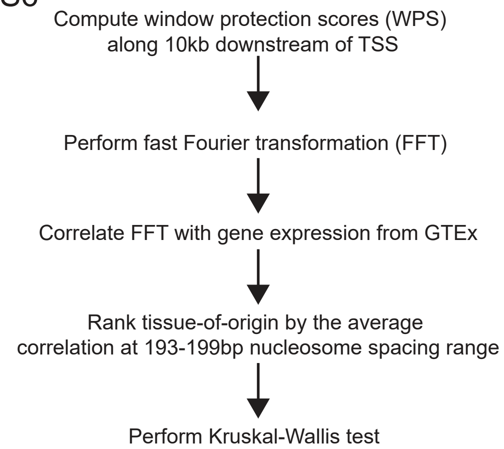

B)

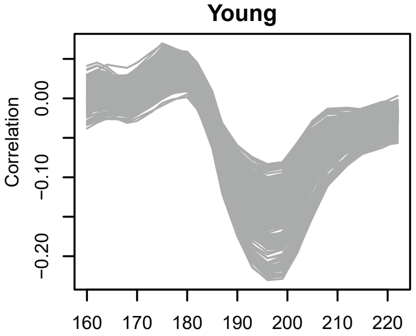

C)

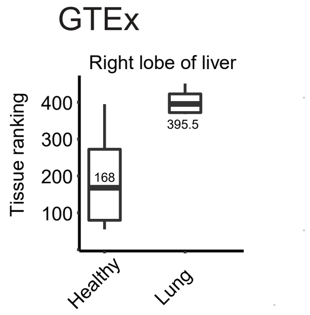

Liver cancer (HCC) - Healthy (adjusted P<0.05)

	Increased in median rank by
Right lobe of liver	**133.5**
Body of pancreas	121
Lymphoblast	32
