# Supplementary material for: Cell‐free DNA as a biomarker of aging
Source: Aging Cell. 2018 Dec 20;18(1):e12890. doi: 10.1111/acel.12890 (PMC6351822; doi:10.1111/acel.12890)
Supplement: Supplementary file 7 [file ACEL-18-e12890-s007.docx]

Table S1


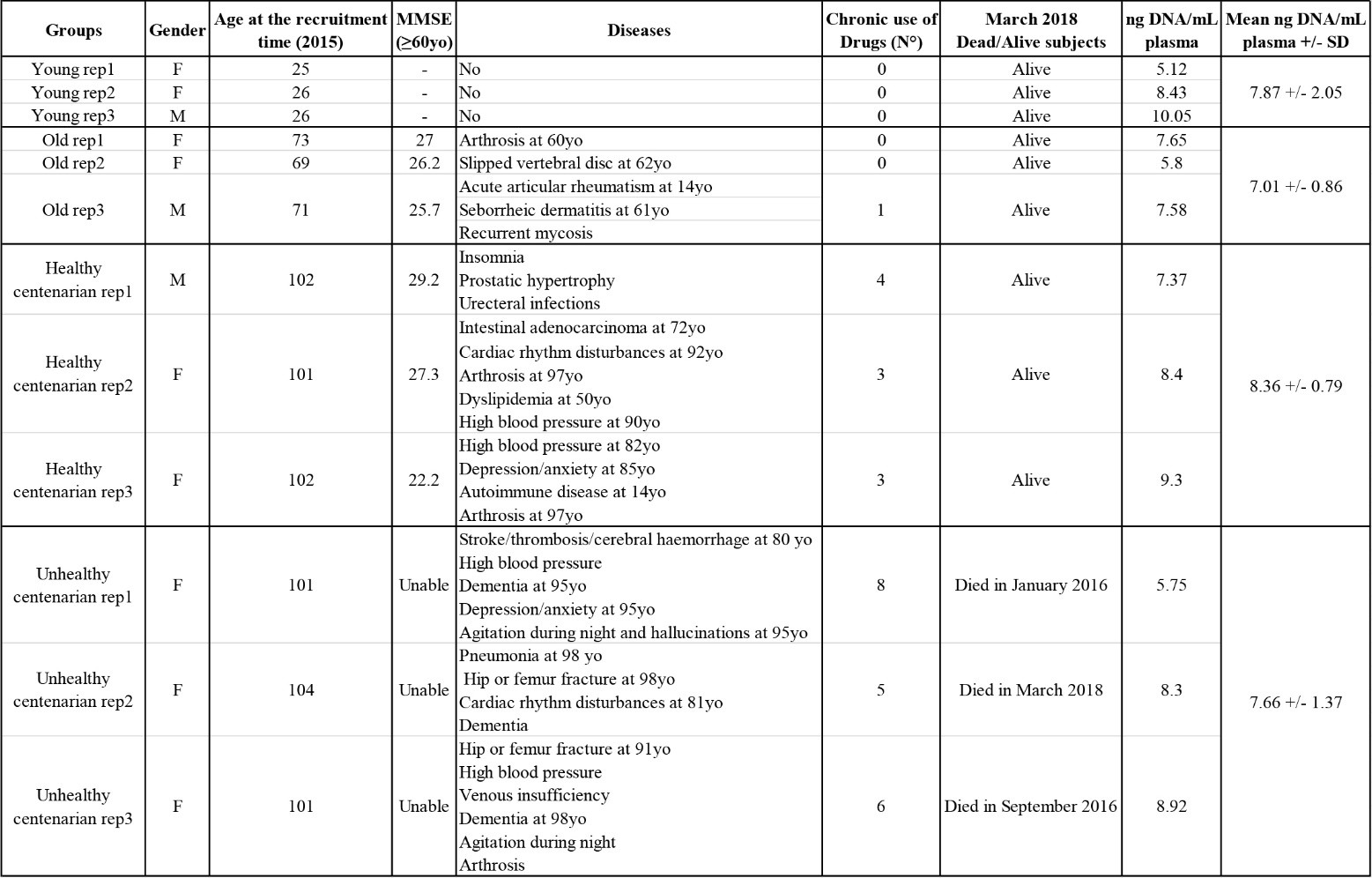


Table S2


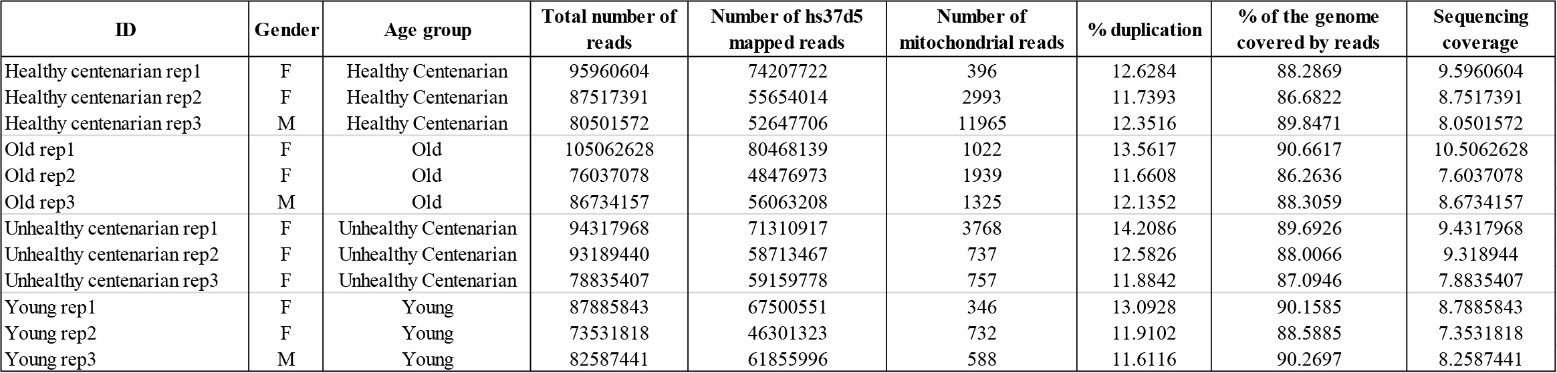


Table S3


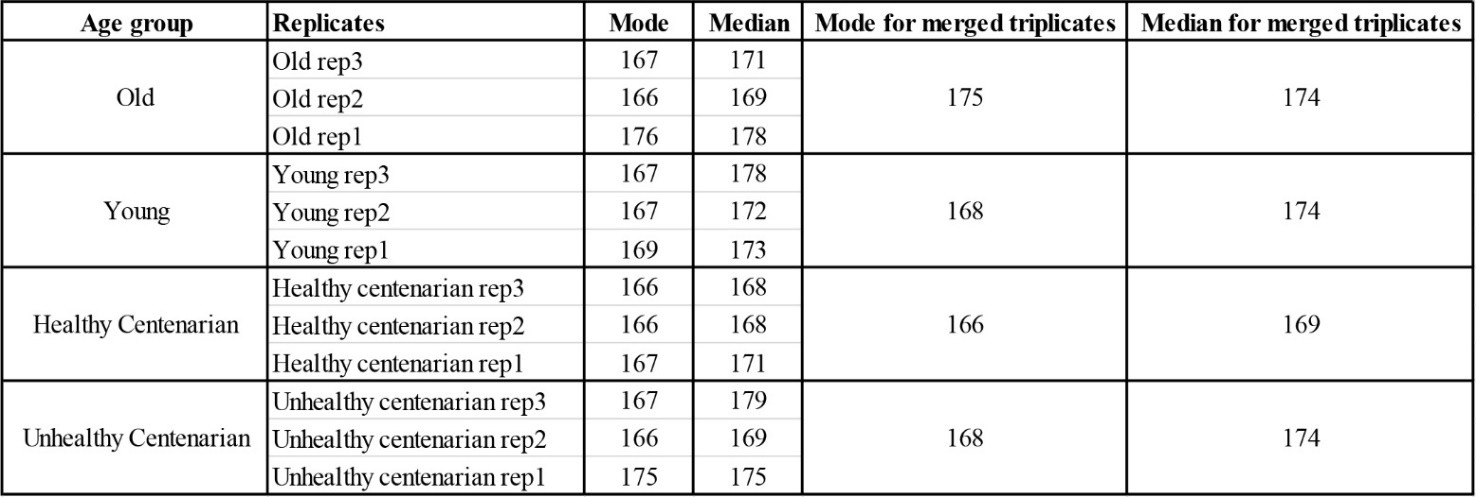


Table S4

|  | **Comparisons** | **Mean of fold change** | **Difference in posterior median** | **Credible interval** | **Change in direction** | **P-value** |
| --- | --- | --- | --- | --- | --- | --- |
| A1 | Healthy centenarian - Old | 0.0447 | 0.0279 | [0.0262, 0.0294] | Increased signal | <0.0001 |
|  | Healthy centenarian - Unhealthy centenarian | 0.0752 | 0.0466 | [0.0451, 0.0483] | Increased signal | <0.0001 |
|  | Healthy centenarian - Young  Old - Unhealthy centenarian | -0.0844  0.0304 | -0.0422  0.0188 | [-0.0458, -0.0425]  [0.0173, 0.0204] | Decreased signal Increased signal | <0.0001  <0.0001 |
|  | Old - Young | -0.1291 | -0.0721 | [-0.0735, -0.0706] | Decreased signal | <0.0001 |
|  | Unhealthy centenarian - Young | -0.1595 | -0.0909 | [-0.0924, -0.0891] | Decreased signal | <0.0001 |
| A2 | Healthy centenarian - Old | 0.0176 | 0.0088 | [0.0078, 0.0097] | Increased signal | <0.0001 |
|  | Healthy centenarian - Unhealthy centenarian | 0.0130 | 0.0041 | [0.0032, 0.0049] | Increased signal | <0.0001 |
|  | Healthy centenarian - Young  Old - Unhealthy centenarian | -0.0165  -0.0046 | 0.0005  -0.0047 | [-0.0005, 0.0014]  [-0.0056, -0.0038] | -  Decreased signal | 0.734  <0.0001 |
|  | Old - Young | -0.0341 | -0.0083 | [-0.0091, -0.0074] | Decreased signal | <0.0001 |
|  | Unhealthy centenarian - Young | -0.0295 | -0.0036 | [-0.0045, -0.0027] | Decreased signal | <0.0001 |
| B1 | Healthy centenarian - Old | -0.0007 | -0.0037 | [-0.0052, -0.0022] | Decreased signal | <0.0001 |
|  | Healthy centenarian - Unhealthy centenarian | 0.0333 | 0.0178 | [0.0164, 0.0192] | Increased signal | <0.0001 |
|  | Healthy centenarian - Young  Old - Unhealthy centenarian | -0.0592  0.0340 | -0.028  0.0216 | [-0.0295, -0.0265]  [0.02, 0.023] | Decreased signal Increased signal | <0.0001  <0.0001 |
|  | Old - Young | -0.0585 | -0.0243 | [-0.0259, -0.0230] | Decreased signal | <0.0001 |
|  | Unhealthy centenarian - Young | -0.0925 | -0.0458 | [-0.0473, -0.0443] | Decreased signal | <0.0001 |
| B2 | Healthy centenarian - Old | -0.0118 | -0.0113 | [-0.0127, -0.01] | Decreased signal | <0.0001 |
|  | Healthy centenarian - Unhealthy centenarian | -0.0179 | -0.0172 | [-0.0184, -0.0158] | Decreased signal | <0.0001 |
|  | Healthy centenarian - Young  Old - Unhealthy centenarian | 0.0077  -0.0061 | 0.0172  -0.0058 | [0.0158, 0.0185]  [-0.0072, -0.0045] | Increased signal Decreased signal | <0.0001  <0.0001 |
|  | Old - Young | 0.0194 | 0.0286 | [0.0272, 0.0299] | Increased signal | <0.0001 |
|  | Unhealthy centenarian - Young | 0.0256 | 0.0344 | [0.033, 0.0356] | Increased signal | <0.0001 |
| B3 | Healthy centenarian - Old | -0.0161 | -0.0144 | [-0.0152, -0.0136] | Decreased signal | <0.0001 |
|  | Healthy centenarian - Unhealthy centenarian | -0.0322 | -0.0272 | [-0.0279, -0.0264] | Decreased signal | <0.0001 |
|  | Healthy centenarian - Young  Old - Unhealthy centenarian | 0.0367  -0.0162 | 0.0373  -0.0128 | [0.0365, 0.0382]  [-0.0135, -0.0120] | Increased signal Decreased signal | <0.0001  <0.0001 |
|  | Old - Young | 0.0529 | 0.0517 | [0.0509, 0.0525] | Increased signal | <0.0001 |
|  | Unhealthy centenarian - Young | 0.0690 | 0.0646 | [0.0637, 0.0653] | Increased signal | <0.0001 |

Table S5

|  | | **RBC (x106/µl)** | **HGB (g/dl)** | **HCT (%)** | **ALB (gr/dl)** | **HDL (mg/100ml)** |
| --- | --- | --- | --- | --- | --- | --- |
| reference range | | 4.20-5.50 | 13-16.5 | 39-54 | 3.50-5.20 | > 39 |
| **HC** | mean  sd | 4.55  0.28 | 13.43  0.29 | 42  0.62 | 4  0.17 | 68  7.55 |
| **UHC** | mean  sd | 3.7  0.32 | 10.53  1.03 | 34.43  3.01 | 3.33  0.12 | 51  5.57 |
|  | p value | **0.025** | **0.009** | **0.013** | **0.005** | **0.035** |

RBC: red blood cell, HGB: hemoglobin, HCT: hematocrit, ALB: albumin, HDL: high density lipoprotein, HC: healthy centenarian, UHC: unhealthy centenarian.
